# Multi-Technique Flavoromics for Identifying Key Differential Volatile Compounds Underlying Sensory Profiles in Lager Beers

**DOI:** 10.3390/foods14193428

**Published:** 2025-10-05

**Authors:** Yiyuan Chen, He Huang, Ruiyang Yin, Xiuli He, Liyun Guo, Yumei Song, Dongrui Zhao, Jinyuan Sun, Jinchen Li, Mingquan Huang, Baoguo Sun

**Affiliations:** 1 China Food Flavor and Nutrition Health Innovation Center, Beijing Technology and Business University, Beijing 100048, China; chenyiyuan_1112@163.com (Y.C.);; 2 Key Laboratory of Brewing Molecular Engineering of China Light Industry, Beijing Technology and Business University, Beijing 100048, China; 3 Beijing Laboratory of Food Quality and Safety, Beijing Technology and Business University, Beijing 100048, China; 4 Technology Center of Beijing Yanjing Beer Co., Ltd., Beijing 101300, China

**Keywords:** lager beer, volatile compounds, odor activity value (OAV), taste activity value (TAV), sensory evaluation, multivariate statistical analysis, flavor addition validation

## Abstract

In this study, inter-brand variations in volatile flavor compound profiles of four lager beers were systematically investigated by integrating sensory evaluation with GC-MS, GC×GC-TOF-MS, and GC-O-MS. A total of 594 volatile compounds were identified, of which 71 with odor activity values (OAV) ≥ 1 were found to contribute directly to aroma expression. Additionally, 59 compounds with taste activity values (TAV) ≥ 1 were identified and may also contribute to taste perception. Furthermore, 53 aroma-active compounds were confirmed through GC-O-MS, providing additional evidence for their sensory contribution. Partial least squares discriminant analysis (PLS-DA), correlation analysis, and flavor addition experiments revealed brand-specific differential flavor compounds. Ultimately, twenty key differential flavor compounds, encompassing esters, alcohols, aromatic compounds, acids, lactones, and others, were confirmed to contribute to fruity, floral, burnt, and sweet notes. Phenethyl alcohol, with concentrations varying from 1377.1 mg/L in QD to 3297.5 mg/L in HR, showed a more than 2.4-fold difference across brands and was strongly associated with fruity (r = 0.553) and floral (r = 0.564) aroma. These compounds acted in combination to shape distinct aroma profiles. This study provides a molecular-level basis for understanding lager beer flavor and offers practical guidance for targeted flavor modulation in brewing.

## 1. Introduction

Lager beer, as one of the most widely consumed beer types globally, owes its market appeal largely to its flavor characteristics, which serve as a decisive factor in consumer preference. It is typically brewed using barley malt as the primary grain source through a multi-stage process involving malting, mashing, wort production, and the addition of hops, along with water and bottom-fermenting yeast [1]. Noticeable differences in flavor are frequently observed among different brands, indicating that the composition and relative concentrations of compounds may play a crucial role in shaping brand-specific profiles. Volatile compounds, including esters, alcohols, and aromatic compounds, are the primary contributors to beer flavor [2], and variations in their types and concentrations directly influence the complexity and uniqueness of the flavor [3]. These compositional differences are largely influenced by raw materials such as malt and hops, brewing processes, and yeast strains [4,5,6]. However, the mechanisms through which these factors drive brand-specific differences in the flavor profile have yet to be systematically elucidated. Although prior studies have explored volatile compound compositions, systematic investigations of the chemical basis and sensory implications of brand-specific differences remain limited, hindering precise flavor modulation to meet growing consumer demand for flavor diversification. In recent years, consumer demand for flavor diversification in beer has been increasing, whereas industrial standardization in production may result in flavor homogenization. Therefore, elucidating inter-brand differences in volatile compounds at the molecular level is of great significance for optimizing flavor modulation and enhancing product competitiveness.

Beer represents a complex matrix rich in proteins and other macromolecules. These constituents often interfere with analytical procedures, leading to column blockage and detector contamination, thereby complicating the reliable extraction and characterization of volatile compounds. Thus, several extraction techniques for volatile compounds in beer have been developed, including liquid–liquid extraction (LLE), headspace solid-phase microextraction (HS-SPME) [7], and solvent-assisted flavor evaporation (SAFE) [8]. The combined application of these methods not only enhances the recovery of volatile compounds with different physicochemical properties but also improves analytical accuracy and representativeness, and they have therefore been widely adopted in current flavor research. Richter et al. compared the effects of HS-SPME and SAFE on hop volatiles, finding that SAFE was more suitable for alcohols and acids, while HS-SPME was suitable for highly volatile compounds such as esters; however, the limited surface area in HS-SPME resulted in a 40% reduction in extracted compounds [9]. In recent years, HS-SPME has become the most widely used extraction technique prior to gas chromatography–mass spectrometry (GC-MS) quantification due to its high efficiency and operational simplicity [10]. HS-SPME has been optimized to efficiently extract a broad spectrum of volatile organic compounds from wort, beer fermentation samples, and finished beer [11]. When combined with GC-MS, HS-SPME has been reported to enable the quantification and annotation of numerous volatile compounds, with up to 397 compounds identified across four types of beer [12]. Nevertheless, GC-MS is subject to co-eluting chromatographic peaks, which can interfere with compound identification, and it is unable to detect certain trace-level constituents. Current analytical techniques often focus on single methods, lacking integration of multiple approaches to comprehensively characterize complex volatile compound profiles and their brand-specific differences. Comprehensive two-dimensional gas chromatography–time-of-flight mass spectrometry (GC×GC-TOF MS), which employs two columns with differing polarities for orthogonal separation, can more effectively resolve compounds with similar retention times and mitigate the impact of co-elution on qualitative and quantitative analyses [13]. A total of 215 volatile metabolites were identified in Undaria-based alcoholic beverages using HS-SPME coupled with GC×GC-TOF-MS. Esters were found to be the most abundant chemical class across all samples, followed by alcohols and ketones, suggesting their predominant contribution to the overall aroma profile [14]. Compared with one-dimensional gas chromatography, GC×GC offers higher peak capacity and sensitivity, and has been widely applied to the analysis of volatile compounds in various foods. However, it does not provide information on the odor characteristics of the compounds or their contribution to the sample aroma [15].

Aroma plays a crucial role in maintaining beer quality and appeal. These flavor compounds typically occur at very low concentrations, accounting for less than 3–5% of the total volatiles in beer [16]. Gas chromatography–olfactometry–mass spectrometry (GC-O-MS) is effective in analyzing aroma-active compounds in complex odor mixtures [17] and has been widely applied in the flavor analysis of various foods. Characteristic aroma-active compounds in wheat beer have been identified using GC-O-MS, including 2-methoxyphenol (smoky, phenolic), 2-ethyl-3,6-dimethylpyrazine (earthy), 2,3-diethyl-5-methylpyrazine (earthy), and maltol (caramel-like) [18]. Among the abundant volatile compounds, only a small subset contributes significantly to beer aroma; these are referred to as key flavor compounds or character-impact aroma compounds [15]. Molecular sensory science has been widely employed to systematically analyze beer flavor and describe key aroma-active compounds [19]. In caramel malt beer, compounds such as (E)-β-damascenone, 2-acetyl-1-pyrroline, methional, 2-ethyl-3,5-dimethylpyrazine, and 6-methyl-5-hepten-2-one have been reported to exhibit particularly high odor activity value (OAV), while 2-methoxyphenol showed the highest OAV in roasted malt beer [20]. However, due to interactions among aroma compounds, even after identifying individual aroma-active compounds, it remains necessary to validate flavor similarity with actual samples using standard references. This is essential for understanding the chemical origins of beer flavor [21]. Furthermore, studies on the correlation between sensory attributes and chemical composition often rely on empirical descriptions, lacking systematic integration based on multi-dimensional data encompassing chemical analysis, sensory evaluation, and statistical modeling.

In this context, the present study focuses on four different brands of lager beer with the objective of systematically elucidating the differences in volatile compounds among brands and revealing their intrinsic associations with sensory attributes through multi-technique integration and multi-dimensional data analysis. Specifically, LLE-SAFE and HS-SPME were combined with GC-MS, GC×GC-TOF MS, and GC-O-MS for comprehensive identification of volatile compounds in lager beers. Molecular sensory science was integrated with multivariate statistical analysis to determine potential differentiating compounds, followed by sensory validation to confirm key differential volatiles. This study provides a chemical basis for brand-specific volatile differences, advancing beer flavor chemistry from empirical description toward precise modulation and supporting product differentiation in a competitive market.

## 2. Materials and Methods

### 2.1. Sample Collection

Four commercially available lager beers were selected for analysis, each representing a distinct brand and formulation. The samples included U8 from Beijing Yanjing Brewery Co., Ltd., Beijing, China, (coded as YJ), Classic from Tsingtao Brewery Group Co., Ltd., Qingdao, China, (coded as QD), Brave the World from China Resources Beer (Holdings) Co., Ltd., Hong Kong, China, (coded as HR), and Ice Beer from Budweiser Asia Pacific Holdings Ltd., Hong Kong, China, (coded as BW). All beers had an original wort concentration of 8 °P and were brewed using water, malt, and hops as the primary ingredients, with variations in adjuncts such as rice, brewing syrup, hop extract, or yeast depending on the brand-specific recipe. These brands were chosen for their extensive consumer base in China, strong market representation, and regional characteristics, reflecting diverse flavor and brewing processes (e.g., variations in raw materials and yeast strains). This selection effectively captures the diversity of brand-specific volatile compounds and regional flavor profiles.

In addition to these commercial samples, a 10 °P lager beer, produced by Beijing Yanjing Brewery Co., Ltd., Beijing, China, under the product name Qing Shuang Beer, was acquired for use as the base matrix in subsequent recombination and addition experiments. This beer was chosen because of its relatively simple volatile background, which minimizes sensory interference. The specific brand formulations are provided in Appendix A Table A1. All samples were purchased from local retail outlets within one week of analysis to minimize storage-related changes in volatile composition. The beers were transported under refrigerated conditions to the laboratory and stored at 4 °C until further processing. One bottle was selected from each brand (YJ, QD, HR, and BW) for analysis. Each beer sample was analyzed in technical triplicate to ensure instrumental reproducibility (*n* = 3).

### 2.2. Materials

Anhydrous ethanol and dichloromethane (99.9%, chromatographic grade) were purchased from Aladdin Biochemical Technology Co., Ltd. (Shanghai, China). Anhydrous sodium sulfate (99.8%, analytical grade) was obtained from Sinopharm Chemical Reagent Beijing Co., Ltd. Internal standards, including 2-ethylbutyric acid (IS1), 4-octanol (IS2), and ethyl valerate (IS3), all of chromatographic grade with a purity of no less than 97.0%, were supplied by J&K Scientific Ltd. (Beijing, China). A mixture of C3–C30 n-alkanes (chromatographic grade) was purchased from Sigma–Aldrich (St. Louis, MO, USA). Analytical standards for the identification and quantification of trace components were obtained from J&K Scientific Ltd. (Beijing, China), all with a purity of at least 97.0%.

### 2.3. LLE-SAFE and HS-SPME

Beer samples were first subjected to liquid–liquid extraction (LLE). A volume of 150 mL of beer was placed in a separation vessel, sodium chloride was added, and the sample was extracted three times with dichloromethane, 50 mL each time. The combined extracts were subsequently processed using solvent-assisted flavor evaporation (SAFE). A 500 mL dry round-bottom flask was used as the receiving vessel, the cold trap was filled with liquid nitrogen, and another round-bottom flask was placed in a water bath maintained at 40 °C. The compound turbomolecular pump was activated, and once the absolute pressure of the system reached 1 × 10^−4^ MPa, the combined LLE extract was slowly introduced into the SAFE apparatus for distillation. The flow rate of the sample was kept constant during operation. The collected distillate was concentrated to 1 mL using a rotary evaporator. All processed samples were stored at −20 °C until subsequent GC-O-MS and GC×GC-TOF MS analyses [22].

To compensate for the loss of low-boiling volatile compounds during LLE, headspace solid-phase microextraction (HS-SPME) was employed for the analysis of volatile components in the beer samples. A 5 mL aliquot of beer was transferred into a 20 mL headspace vial, followed by the addition of 25 μL of internal standard solution (2-ethylbutyric acid, IS1; 4-octanol, IS2; ethyl valerate, IS3) and 1.50 g of sodium chloride to saturation. The vial was immediately sealed with a silicone septum cap. The sample was equilibrated in a water bath at 40 °C for 30 min, after which a 50/30 μm DVB/CAR/PDMS fiber was exposed to the headspace for 40 min at the same temperature. The fiber was then promptly inserted into the gas chromatograph injection port and desorbed at 250 °C for 5 min for analysis [23,24].

### 2.4. Analytical Instrumentation and Conditions

#### 2.4.1. GC-MS Conditions

Volatile compounds were analyzed using a GC-MS system equipped with a polar DB-FFAP column (60 m × 0.25 mm × 0.25 μm). Each sample (1.0 μL), prepared via HS-SPME, was injected in splitless mode, and each analysis was performed in triplicate. High-purity helium (99.999%) was used as the carrier gas at a constant flow rate of 1.0 mL/min. The injector temperature was set at 250 °C. The column temperature program was as follows: initial temperature of 40 °C held for 5 min, increased at 5 °C/min to 70 °C and held for 15 min, then increased at 1 °C/min to 100 °C and held for 5 min, followed by an increase at 3 °C/min to 240 °C and held for 10 min. The mass spectrometer was operated in electron impact (EI) mode at 70 eV, with quadrupole and ion source temperatures set at 150 °C and 230 °C, respectively. The scan range was 35–550 amu in full-scan mode, with a solvent delay of 3 min and a transfer line temperature of 245 °C.

#### 2.4.2. GC-O-MS Conditions

GC-O-MS was used to identify potential aroma-active compounds. The DB-FFAP column (60 m × 0.25 mm × 0.25 μm) was used with helium (99.999%) as the carrier gas at a flow rate of 1.0 mL/min. Samples (1.0 μL) were injected in splitless mode, and the effluent was split 1:1 between the mass spectrometer (250 °C) and the olfactometry port (250 °C). Three trained panelists (one male and two females) performed GC-O analysis. During operation, panelists positioned their noses above the olfactometry port to sniff and record the retention time, odor description, and intensity of each eluted compound. Odor intensity was rated on a five-point scale (0 = none, 1 = very weak, 2 = weak, 3 = moderate, 4 = strong, 5 = very strong), and the Osme value for each compound was calculated as the average score from the three panelists. Each sample was analyzed in triplicate. The column oven was initially held at 40 °C, increased at 10 °C/min to 50 °C and held for 20 min, then increased at 1 °C/min to 70 °C and held for 10 min, and finally increased at 3 °C/min to 250 °C and held for 15 min. The mass spectrometer operated in EI mode at 70 eV in full-scan mode over the range *m*/*z* 45–550 amu [25].

#### 2.4.3. GC×GC-TOF MS Conditions

A GC×GC-TOF MS system was used for the analysis of trace volatile compounds. The system was equipped with two columns: a DB-Wax column (60 m × 0.25 mm × 0.25 μm) as the first dimension and a DB-17 ms column (1.85 m × 0.18 mm × 0.18 μm) as the second dimension, with a thermal modulator positioned between the columns. Helium (99.999%) was used as the carrier gas at a constant flow rate of 2.1 mL/min. The primary oven temperature program was as follows: initial temperature of 40 °C held for 5 min, increased at 3 °C/min to 230 °C and held for 10 min. The cold zone of the modulator was set to −50 °C. The hot and cold jet temperatures were maintained at 70 °C and 160 °C above the primary oven temperature, with maximum temperatures of 260 °C and 320 °C, respectively. The modulation period was 5 s. For TOF MS detection, the transfer line and ion source temperatures were set to 250 °C and 230 °C, respectively, with electron impact energy at 70 eV. The mass range was *m*/*z* 35–350 amu [26].

### 2.5. Qualitative and Quantitative Analysis

The qualitative identification of volatile compounds in beer was performed using four criteria. For mass spectrometric (MS) identification, background noise was subtracted from chromatograms, and compound spectra were matched against the NIST 2020 library and the LIQUOR (in-house) mass spectral libraries. Only compounds with a match score greater than 80% were retained. For retention index (RI) identification, n-alkanes (C_3_–C_30_) were analyzed before and after sample injection under identical chromatographic conditions, and retention indices were calculated based on retention times. Compounds with RI values within an absolute difference of 30 from literature data were considered identical. For aroma sniffing identification, compounds whose odor characteristics matched those of the reference standards were considered identical. For standard substance identification, standards were analyzed under the same chromatographic conditions as the beer samples. Compounds were confirmed as identical when their characteristic ion peaks and retention times were consistent between the sample and the standard mass spectra.

Quantification of volatile compounds was performed using internal standards in combination with calibration curves. A 3% (*v*/*v*) aqueous ethanol solution was used as the matrix, and working standard solutions were prepared at appropriate gradient concentrations based on the levels of each compound in beer. Both the working standards and beer samples were analyzed under the same chromatographic conditions. Calibration curves were constructed by plotting the ratio of the peak area of the compound to that of the internal standard on the *x*-axis against the compound concentration on the *y*-axis. Internal standard IS1 was used for acids, IS2 for alcohols, and IS3 for esters and other compounds. Calibration curve information is shown in Appendix A Table A2. All analyses were performed in triplicate [27].

### 2.6. Odor Activity Value (OAV) and Taste Activity Value (TAV) Calculation

Odor activity values (OAV) and taste activity values (TAV) are defined as the ratios of compound concentrations to their respective odor and taste thresholds [27]. Based on the quantitative results and the published sensory thresholds for each compound in alcoholic beverages, OAV and TAV values were calculated for trace compounds in all representative samples [28,29].

### 2.7. Flavor Perception Evaluation of Lager Beers

Prior to the experiment, ten trained panelists (five males and five females, aged 21–28 years) with at least one year of experience in sensory evaluation were recruited from the Key Laboratory of Brewing Molecular Engineering of China Light Industry to form a sensory evaluation panel. The sensory quantitative descriptive analysis (SQDA) method was applied to assess the sensory attributes of the beer samples. All panelists completed a two-week structured training program, which included aroma reference compound familiarization, terminology alignment, intensity scaling practice using representative beer samples, and consensus development on aroma descriptors. Frequently occurring descriptive terms were screened and compiled into a standardized list of sensory attributes, based on both training sessions and the aroma descriptions of the beers. The final set of sensory attributes included fruity, floral, woody, husky, grassy, cereal-like, roasted, phenolic, acidic, sulfurous, oily, sweet, and off-flavor notes.

Additionally, three panelists, selected from the SQDA panel based on specialized olfactory training, formed the gas chromatography–olfactometry (GC-O) panel to identify key aroma-active compounds. The smaller GC-O panel size was due to the high level of specialized olfactory expertise required, typically performed by a limited number of highly trained experts to ensure precise olfactory data, whereas SQDA required a larger panel to capture the diversity and statistical significance of sensory attributes [30,31].

Sensory evaluations were conducted in a controlled environment (25 ± 1 °C and 35–50% relative humidity) within isolated booths to prevent panelist interaction. Each sample (50 mL) was presented in a 100 mL glass bottle coded with a random three-digit number, and each sample was evaluated in triplicate by every panelist (*n* = 3). The panelists were instructed to rate the intensity of each sensory attribute on a 10-point scale (0 = none, 5 = moderate, 10 = very strong). Sample presentation order was randomized to minimize order effects, and all evaluations were carried out under uniform lighting and ventilation to reduce contextual bias.

To assess whether compounds with an odor activity value (OAV) ≥ 1 are sufficient to reproduce the aroma sensory characteristics of different lager beer brands, the set of OAV ≥ 1 compounds identified in each of the four beers was selected as the aroma-active component pool for recombination modeling. A 3% (*v*/*v*) aqueous ethanol solution was used as the model base, and the selected aroma-active compounds were incorporated at their experimentally determined concentrations. After equilibration at 25 ± 1 °C for 20 min to ensure homogeneity, four recombined beer aroma models were prepared. Each recombined model and its corresponding original beer sample were evaluated following the same procedure described above, using identical sample presentation, coding, evaluation environment, and scoring method [32].

Although the sensory panel underwent rigorous training to ensure consistency, the small GC-O panel size (3 panelists) may limit the diversity of olfactory data, and the SQDA panel (10 panelists) and its young age range (21–28 years) may not fully reflect the flavor preferences of a broader consumer population.

### 2.8. Adding Validation Experiment

An addition experiment was conducted to verify the contribution of the screened key differential volatile compounds to the aroma sensory attributes of lager beer [33]. Yanjing Qing Shuang Beer was selected as the base matrix due to its relatively simple volatile background, which minimizes sensory interference and allows clearer identification of the effects of the added compounds. Based on the actual concentrations determined in the tested samples, twenty key differential volatiles were selected and grouped into ten categories according to their chemical classifications: esters, alcohols, aromatics, acids, furans, lactones, sulfur-containing compounds, aldehydes, ketones, and others. This classification strategy, widely applied in sensory science, was adopted to facilitate systematic evaluation and to minimize interpretive bias from cross-category interactions.

All compounds were incorporated into the base matrix at their respective concentrations as measured in the original beer samples. No sensory-based concentration adjustments were made, ensuring that the assessment reflected real-world exposure conditions and enabling objective comparison of class-specific aroma contributions. The spiked samples were evaluated by the same panel of ten trained assessors following the procedure described in Section 2.7.

### 2.9. Statistical Analysis and Statistical Methods

Statistical analyses were conducted using Excel and IBM SPSS Statistics 26.0 (Chicago, IL, USA). Pearson correlation coefficients were calculated for correlation analysis. A *p*-value of less than 0.05 was considered statistically significant. Basic data processing was performed in Excel. The results were expressed as mean ± standard deviation (SD), based on three parallel replicates per sample. Data visualization was carried out using Origin 2021 (OriginLab Co., Northampton, MA, USA). Partial least squares discriminant analysis (PLS-DA) was performed using SIMCA 14.1 (Umetrics, Umeå, Sweden) to discriminate among beer samples based on volatile compound profiles and to identify variables contributing most to group separation.

## 3. Results and Discussion

### 3.1. Analysis of Distribution Characteristics of Volatile Compounds

**Figure 1:**
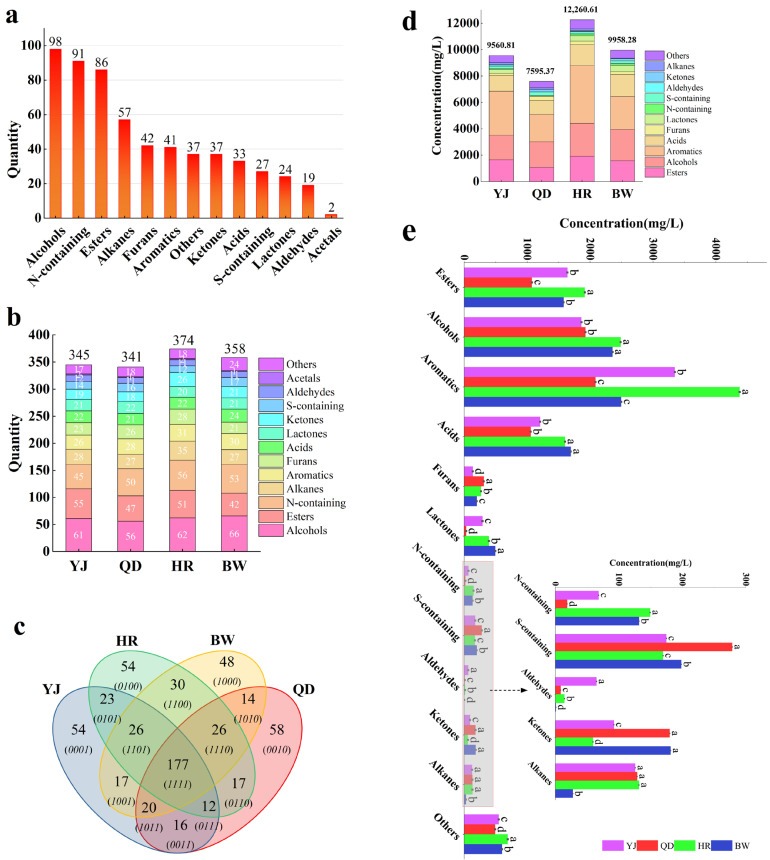
(**a**) Bar chart of identified volatile compounds in different brands of lager beer. (**b**) Stacked bar chart of compound classes in different brands of lager beer. (**c**) Venn diagram of compound classes identified in different brands of lager beer. (**d**) Stacked bar chart of total concentrations of volatile compounds in different brands of lager beer. (**e**) Bar chart of concentrations of volatile compounds in different brands of lager beer. The letters a, b, c, and d represent concentrations with significant differences (*p* < 0.05). The average concentration is highest for letter a and lowest for letter d. Abbreviations YJ, QD, HR, and BW are defined in the manuscript’s Abbreviations section: YJ (U8, Beijing Yanjing Brewery Co., Ltd.), QD (Classic, Tsingtao Brewery Group Co., Ltd.), HR (Brave the World, China Resources Beer (Holdings) Co., Ltd.), and BW (Ice Beer, Budweiser Asia Pacific Holdings Ltd.).

Differences in the distribution of compounds among lager beers from different brands were investigated through qualitative analysis, as illustrated in Figure 1a,b. Although the beers originated from different manufacturers, their volatile profile exhibited a similar distribution pattern, dominated by alcohols, esters, and nitrogen-containing compounds. Alcohols were the most diverse group, primarily generated through microbial metabolism and serving as precursors for ester formation. Overall, the numbers of compounds identified in YJ, QD, HR, and BW were 345, 341, 374, and 358, respectively, with HR showing the highest number, followed by BW (Appendix A Table A3).

Despite the comparable total numbers and categories of compounds, the Venn diagram in Figure 1c revealed significant brand-specific differences. Across the four brands, 177 volatile compounds were shared, while YJ, QD, HR, and BW contained 54, 58, 54, and 48 unique compounds, respectively. Examples of unique compounds include β-butyrolactone, ethyl thioacetate, 3-methylbutanal, ethyl benzoate, nerolidyl formate, and 1-heptanol in YJ; 1,2-butanediol, ethyl 3-methylbutanoate, butyl butanoate, butanoic acid, and 5-methyl-3-heptanone in QD; 2,5-dimethylphenol, 2-n-butylfuran, 5-methyl-2-furfural, and methyl lactate in HR; and resorcinol, ethyl cinnamate, 4-methylpentanoic acid, nonanoic acid, and methyl formate in BW. These compounds exhibit diverse sensory properties and interact with one another, collectively shaping the complexity of lager beer aroma.

Aroma-active compounds were further characterized using LLE-SAFE and gas chromatography–olfactometry (GC-O). In total, 53 aroma compounds were detected across the four beers, comprising 9 aromatics, 9 acids, 7 nitrogen-containing compounds, 7 esters, 5 furans, 4 alkanes, 4 alcohols, 3 lactones, 2 ketones, 2 sulfur-containing compounds, and 1 miscellaneous compound (Appendix A Table A4). Among these, 21 compounds were consistently identified as aroma-active in all beers, including 4-vinylguaiacol, γ-decalactone, phenylacetic acid, 3-methylbutanoic acid, sotolon, 2-phenylethyl acetate, phenylethanol, hexanoic acid, and 1-octen-3-ol, which were deemed as key contributors to the typical sensory profile of lager beers. Acids imparted sour and dark wheat-like notes, such as cis-vaccenic acid and palmitic acid, while some, like acetic acid, produced undesirable sour and muddy odors. Esters generally provided sweet, fruity, and floral notes, with monoethyl succinate contributing malty aromas. Alcohols mainly presented roasted, dark wheat, and husky notes, whereas aromatics, lactones, and ketones often offered sweet and floral attributes, such as γ-decalactone, which imparted gardenia-like sweetness and creamy notes. Most furans gave unpleasant aromas, although sotolon was an exception, imparting a pleasant caramel sweetness.

For quantitative analysis, 150 compounds were selected based on their strong aroma relevance, including 36 esters, 23 alcohols, 19 aromatics, 19 acids, 11 furans, 10 lactones, 9 nitrogen-containing compounds, 9 sulfur-containing compounds, 6 others, 4 aldehydes, 3 ketones, and 1 alkane. Significant differences (*p* ≤ 0.05) in the concentrations of individual compounds among the beers were observed, such as ethyl isovalerate, ethyl propanoate, hexyl butanoate, ethyl lactate, γ-decalactone, and furfuryl alcohol. The total concentration of volatile compounds was highest in HR (12260.61 mg/L), followed by BW (9958.28 mg/L), YJ (9560.81 mg/L), and QD (7595.37 mg/L), consistent with their compositional diversity. Esters, alcohols, aromatics, and acids were the dominant quantitative contributors. Ethyl acetate, a major ester, showed the highest concentration among esters in YJ (554.93 ± 7.64a mg/L) and QD (449.33 ± 9.29b mg/L), and was also abundant in HR (515.28 ± 52.73a mg/L) and BW (367.56 ± 6.76c mg/L), second only to ethyl 3-methylbutyrate in the latter two [34]. Ethyl 3-methylbutanoate, known to enhance fruity aromas, was abundant in all beers but declines during storage, potentially impacting flavor stability [35].

Figure 1e illustrated that aromatic compounds contributed most to the between-brand differences, with HR containing the highest total aromatic content (4377.93 mg/L), followed by YJ (3348.73 mg/L), BW (2495.21 mg/L), and QD (2087.12 mg/L). HR also had the highest total concentrations of esters (1917.86 mg/L), alcohols (2485.89 mg/L), and nitrogen-containing compounds (149.26 mg/L), whereas BW contained the highest total acids (1692.25 mg/L) and lactones (493.46 mg/L), and QD exhibited the highest total furans (311.13 mg/L), sulfur-containing compounds (278.57 mg/L), and ketones (180.07 mg/L). Notably, 2-phenylethanol was the most abundant volatile in all beers, with concentrations of 3297.47 ± 217.49a, 2374.06 ± 285.93b, 1760.47 ± 226.12c, and 1377.05 ± 303.76c mg/L in HR, YJ, BW, and QD, respectively. Other abundant volatiles included 3-methyl-1-butanol, ethyl acetate, glycerol, 2,6-di-tert-butyl-p-cresol, 1-heptene, octanoic acid, ethyl 3-methylbutanoate, and 1-pentanol. However, due to matrix effects, the contribution of each compound to beer flavor cannot be determined solely based on concentration.

### 3.2. Flavor Expression Evaluation

In order to screen the flavor compounds, odor specific magnitude estimation (Osme), odor activity values (OAV), and taste activity values (TAV) were applied to evaluate flavor expression for volatile compounds.

**Figure 2:**
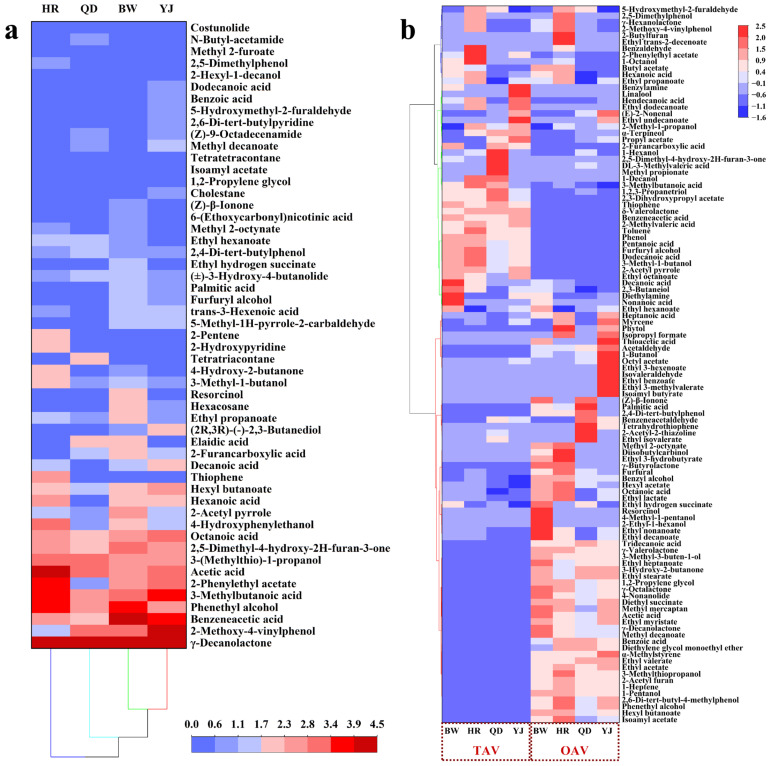
(**a**) Heatmap of Osme results for different brands of lager beer. (**b**) Heatmap of compounds with TAV/OAV ≥ 1. A redder color indicates a higher value. Abbreviations YJ, QD, HR, and BW are defined in the manuscript’s Abbreviations section.

The Osme method was applied to record the aroma intensity of the aroma-active compounds, and the results were visualized as a heatmap (Figure 2a). Overall, acids, aromatics, and lactones exhibited higher aroma intensities than other classes of aroma-active compounds, followed by furans. Differences in aroma intensity among compounds were observed across the lager beer samples. In YJ, the most intense compounds were γ-decalactone (2.94 ± 0.05 mg/L), 4-vinylguaiacol (23.35 ± 0.29 mg/L), phenylacetic acid (101.22 ± 6.24 mg/L), 3-methylbutanoic acid (18.32 ± 2.46 mg/L), 2-phenylethyl acetate (25.76 ± 3.66 mg/L), acetic acid (17.47 ± 4.32 mg/L), and octanoic acid (413.13 ± 46.23 mg/L). In BW, γ-decalactone (8.77 ± 0.38 mg/L), phenylacetic acid (91.45 ± 3.00 mg/L), and 2-phenylethanol (1760.47 ± 226.12 mg/L) were dominant. In HR, the highest intensities were for γ-decalactone (5.15 ± 0.20 mg/L), acetic acid (12.29 ± 1.60 mg/L), 3-methylbutanoic acid (34.84 ± 1.53 mg/L), 2-phenylethyl acetate (59.37 ± 3.69 mg/L), and 2-phenylethanol (3297.47 ± 217.49 mg/L). In QD, γ-decalactone (3.63 ± 0.43 mg/L), 4-vinylguaiacol (24.23 ± 0.80 mg/L), acetic acid (9.53 ± 2.05 mg/L), and 3-methylbutan-1-ol (421.12 ± 70.96 mg/L) were most pronounced. Compared to YJ, BW showed a 198.30% increase in γ-decalactone concentration, a 9.66% decrease in phenylacetic acid, and a 25.86% decrease in 2-phenylethanol. Relative to YJ, HR exhibited a 75.17% increase in γ-decalactone, a 29.66% decrease in acetic acid, a 90.17% increase in 3-methylbutanoic acid, a 130.51% increase in 2-phenylethyl acetate, and a 38.90% increase in 2-phenylethanol. In comparison to YJ, QD demonstrated a 23.47% increase in γ-decalactone, a 3.77% increase in 4-vinylguaiacol, a 45.45% decrease in acetic acid, and a 25.23% decrease in 3-methylbutan-1-ol. γ-Decalactone consistently showed the highest aroma intensity in all four beers, suggesting its potential importance as a key aroma-active compound in lager beer. Other compounds exhibited sample-specific variations without a consistent trend.

As previously noted, the sensory contribution of volatile compounds in beer depends not only on their concentrations but also on their interactions and the matrix effect. Although GC-O analysis is an effective method for identifying odorants, it does not account for the influence of the beer matrix [25]. To assess the contribution of volatile compounds to lager beer flavor, odor activity values (OAVs) were calculated based on literature-reported aroma thresholds. Compounds with OAV ≥ 1 were considered to contribute directly to beer flavor, and a higher OAV indicated a greater contribution to overall flavor quality. The results (Table A5) showed that a total of 71 flavor compounds with OAV ≥ 1 were identified across the four lager beers, including 21 esters, 11 alcohols, 11 aromatics, 9 acids, 4 furans, 4 lactones, 4 sulfur-containing compounds, 3 aldehydes, 2 ketones, and 2 others. YJ, QD, HR, and BW contained 60, 52, 57, and 55 such compounds, respectively. Among these, 44 compounds were common to all four beers, mainly esters, acids, and aromatics. Acids were primarily associated with cheesy notes, esters were the major flavor contributors in beer with floral and fruity characteristics, and aromatics imparted rose-like notes [3,25]. These findings suggest that esters, acids, and aromatics are the most important flavor groups in lager beer.

The highest OAV differed among samples. In YJ, 3-methylbutyl acetate (2,046,526) ranked first, followed by hexanoic acid (258,687), trans-2-nonenal (149,938), methyl decanoate (19,448), 2-methyl-1-propanol (14,796), and ethyl acetate (11,099). In QD, the top compounds were (Z)-β-ionone (14,243,050), 3-methylbutyl acetate (1,217,009), hexanoic acid (198,095), trans-2-nonenal (41,011), 3-methylbutyl propanoate (31,502), methyl decanoate (16,822), and 2-methyl-1-propanol (12,246). In HR, 3-methylbutyl acetate (4,338,735) was the highest, followed by hexanoic acid (474,974), methyl decanoate (29,701), 2-methyl-1-propanol (15,732), and ethyl acetate (10,306). In BW, (Z)-β-ionone (12,784,795) ranked first, followed by 3-methylbutyl acetate (2,821,490), hexanoic acid (419,423), methyl decanoate (45,970), and ethyl hexanoate (10,366). Some compounds, such as (Z)-β-ionone, 3-methylbutyl propanoate, hexanoic acid, methyl decanoate, and ethyl acetate, consistently exhibited high OAV in all samples, indicating substantial contributions to lager beer aroma. Certain low-abundance compounds, including myrcene, methanethiol, γ-decalactone, 4-vinylguaiacol, phenylacetaldehyde, and 3-methylbutan-1-ol, also had OAV ≥ 1 due to low aroma thresholds, whereas some high-abundance compounds, such as glycerol, 5-hydroxymethyl-2-furfural, monoethyl succinate, and diethyl succinate, showed low OAV due to high thresholds. Overall, these 71 high OAV compounds may constitute the core flavor framework of lager beer.

Odor and taste are two primary drivers of flavor perception that can act independently or interact through cross-modal effects. One such interaction is odor-induced taste perception (OICTP), in which volatile compounds stimulate olfactory receptors in the nasal epithelium retronasally during consumption, altering the taste sensations perceived by the taste buds [36,37]. Taste activity values (TAV) were calculated for volatile compounds, with TAV ≥ 1 indicating a direct contribution to beer taste. The results (Table A5, Figure 2b) showed that 59 compounds had TAV ≥ 1, including 18 esters, 9 alcohols, 9 aromatics, 9 acids, 4 lactones, 4 sulfur-containing compounds, 2 furans, 2 aldehydes, 1 ketone, and 1 other. Among these, 37 were present with TAV ≥ 1 in all four beers. The numbers of compounds with TAV ≥ 1 were 49, 46, 51, and 43 in YJ, QD, HR, and BW, respectively. In YJ, the highest TAV was for hexanoic acid (279,058), followed by trans-2-nonenal (113,953) and 3-methylbutyl acetate (102,326). In QD, hexanoic acid (213,694), 2-acetyl-2-thiazoline (70,957), and 3-methylbutyl acetate (60,850) were highest. In HR, hexanoic acid (512,378), 3-methylbutyl acetate (216,937), and 2-methyl-1-propanol (19,592) ranked highest. In BW, hexanoic acid (452,452), 3-methylbutyl acetate (141,074), and ethyl octanoate (18,693) were most pronounced. Hexanoic acid and 3-methylbutyl acetate thus appear to be the major contributors to taste perception in lager beer. Previous studies have shown that hexanoic acid enhances beer sourness and prolongs its perception, which can affect bitterness perception after swallowing [38]. 3-methylbutyl acetate imparts banana-like and ester aromas, and at higher concentrations it can mask the flavor expression of other compounds, such as 2-methylbutanal, 3-(methylthio)propanal, and phenylacetaldehyde [39]. A total of 33 flavor compounds were identified with both an odor activity value (OAV) ≥ 1 and a taste activity value (TAV) ≥ 1 across the four types of lager beer, including 3-methylbutyl acetate, ethyl hexanoate, 2-methyl-1-propanol, hexanoic acid, γ-decalactone, 2-phenylethanol, 6-methyl-5-hepten-2-one, ethyl pyruvate, among others. These compounds are likely to serve as key contributors to the characteristic flavor profile of lager beers.

### 3.3. Flavor Perception Evaluation

**Figure 3:**
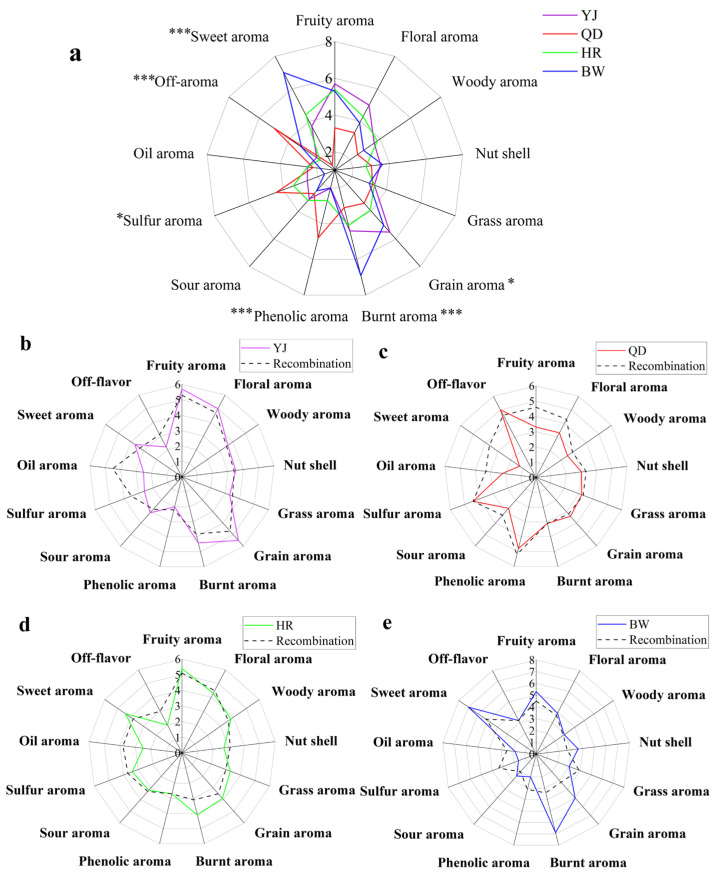
(**a**) Aroma sensory radar chart of different brands of lager beer. Asterisks * and *** indicate significant differences at *p* ≤ 0.05 and *p* ≤ 0.001, respectively. (**b**–**e**) Comparison of aroma recombination models and original beer samples for lager beer. Radar charts correspond to YJ, QD, HR, and BW, respectively. Abbreviations YJ, QD, HR, and BW are defined in the manuscript’s Abbreviations section.

To investigate the aroma sensory characteristics of different lager beer brands, sequential quantitative descriptive analysis (SQDA) was conducted to assess the aroma profiles and intensity of four beer samples, followed by analysis of variance (Duncan’s test) to determine significant differences in aroma attributes. As shown in Figure 3a, the overall aroma profiles of the four beers exhibited notable differences. Significant differences (*p* ≤ 0.001) were observed in sweet aroma, burnt aroma, phenolic aroma, and off-flavor attributes, while sulfur aroma and grain aroma differed significantly (*p* ≤ 0.05). No significant differences were found for fruity aroma, floral aroma, woody aroma, nut shell, grass aroma, sour aroma, and oil aroma. Specifically, the YJ sample was characterized by pronounced grain aroma and fruity aroma, with noticeable floral aroma, sweet aroma, and burnt aroma; the QD sample showed prominent phenolic aroma, sulfur aroma, and fruity aroma, along with a distinct off-flavor; the HR sample exhibited strong fruity aroma and sweet aroma, with marked floral aroma and burnt aroma; the BW sample displayed intense sweet aroma and burnt aroma, followed by fruity aroma and grain aroma. These differences are closely related to the composition, concentration, and interactions of volatile compounds, indicating the necessity of molecular-level investigation into their sensory contributions.

Based on these findings, flavor compounds with OAV ≥ 1 were blended according to their concentrations in the four lager beer samples to prepare simulated recombination models, which were subsequently evaluated by SQDA. The sensory panel assessed the intensity of 13 aroma attributes (fruity aroma, floral aroma, woody aroma, nut shell, grass aroma, grain aroma, burnt aroma, phenolic aroma, sour aroma, sulfur aroma, oil aroma, sweet aroma and off-flavor) for both the original samples and their corresponding recombination models. Results indicated that the aroma profiles of the recombination models were highly similar to those of the original beers, with Pearson correlation coefficients of 0.78, 0.68, 0.74, and 0.64 for YJ, QD, HR, and BW, respectively. As illustrated in Figure 3b, the YJ recombination model successfully reproduced the dominant grain aroma and fruity aroma, as well as distinct floral aroma, sweet aroma, and burnt aroma, showing good similarity in woody aroma, nut shell, grass aroma, phenolic aroma, and sour aroma. Figure 3c shows that the QD model effectively reproduced the overall aroma profile of the original beer, with a slight enhancement in sweet aroma. Figure 3d indicates that the HR model achieved a high level of similarity to the original. Figure 3e demonstrates that the BW model reproduced most of the original aroma characteristics, although a deviation was noted in burnt aroma intensity.

These results suggest that the primary aroma characteristics of lager beer are shaped by the synergistic effects of the selected flavor compounds. However, despite the overall similarity between the recombination models and the original samples, subtle differences in certain sensory attributes were noted by the panel. These discrepancies may be attributed to the absence of compounds with odor activity values (OAVs) below 1 or non-volatile matrix components that were not included in the models [40]. Notably, in complex multicomponent systems such as beer, aroma perception arises not only from the intensity of individual volatiles but also from their perceptual interactions. Compounds present at sub-threshold levels can influence the perception of other aroma-active compounds through mechanisms such as synergistic enhancement, blending, or masking, thereby contributing to the overall realism and complexity of the aroma profile. Therefore, to improve the fidelity of aroma reproduction, future research should consider incorporating low-OAV compounds into reconstitution models or conducting targeted omission/addition experiments to better understand their functional roles in sensory perception.

### 3.4. Screening of Potential Differential Compounds by PLS-DA

To identify the key factors contributing to sensory differences among different lager beer brands, supervised partial least squares discriminant analysis (PLS-DA) was employed to extract informative variables from the flavor compound dataset. PLS-DA is a multivariate statistical method that performs dimensionality reduction while establishing a relationship model between variables and sample categories, thereby effectively separating samples in the reduced dimensional space.

**Figure 4:**
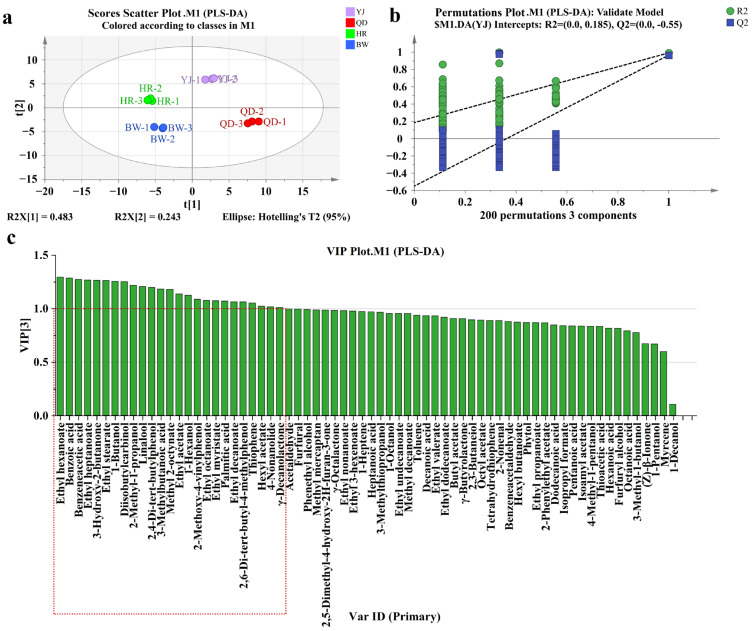
PLS-DA analysis of different brands of lager beer. (**a**) Score scatter plot. (**b**) Permutation test plot. (**c**) VIP score plot. Abbreviations YJ, QD, HR, and BW are defined in the manuscript’s Abbreviations section. The red dotted box highlights compounds with a Variable Importance in Projection (VIP) score greater than 1.

To ensure statistical reliability and prevent overfitting, we conducted a 200-iteration permutation test for model validation. As shown in Figure 4a, the four lager beer samples were clearly separated in the two-dimensional score plot. The first and second principal components (R^2^X [1] and R^2^X [2]) explained 0.483 and 0.243 of the total variance, respectively. The model’s explained variance (R^2^Y) and predictive ability (Q^2^Y) were 0.993 and 0.981, indicating strong explanatory and predictive power. Permutation test results (Figure 4b) showed that all permuted R^2^ and Q^2^ values were lower than those of the original model, and the intercept of the Q^2^ regression line on the vertical axis was below zero (Q^2^Y = –0.55). These results confirm that the PLS-DA model is statistically robust and free from overfitting.

Variable importance in projection (VIP) scores were calculated to evaluate the contribution of each flavor compound to sample discrimination. Following widely accepted chemometric standards, variables with VIP > 1 were considered significant contributors to inter-brand differentiation [41,42]. As shown in Figure 4c, 25 flavor compounds had VIP > 1, including ethyl hexanoate (VIP = 1.29432), phenylacetic acid (VIP = 1.27321), diacetyl (VIP = 1.26486), 2-methyl-1-propanol (VIP = 1.21913), linalool (VIP = 1.20656), 3-methylbutanoic acid (VIP = 1.18217), ethyl acetate (VIP = 1.13657), 4-vinylguaiacol (VIP = 1.08803), δ-nonalactone (VIP = 1.01653), and γ-decalactone (VIP = 1.01045). Among these, ethyl hexanoate, 4-vinylguaiacol, phenylacetic acid, 3-methylbutanoic acid, γ-decalactone, δ-nonalactone, and diacetyl had previously been identified as aroma-active compounds in the GC-O analysis, suggesting their potential role as key characteristic flavor compounds in lager beer. Nevertheless, these findings derived from mathematical modeling require further validation through sensory evaluation.

### 3.5. Validation Experiment Analysis

Given the discrepancies among the OAV analysis, Osme results, and PLS-DA outcomes, a comprehensive evaluation was performed to identify the key differential flavor compounds influencing aroma expression in different lager beer brands. A total of 20 compounds were selected, including two esters (ethyl hexanoate, ethyl acetate), two alcohols (2-methyl-1-propanol, linalool), five aromatic compounds (phenethyl alcohol, 2-phenylethyl acetate, benzeneacetaldehyde, 2-methoxy-4-vinylphenol, benzeneacetic acid), four acids (octanoic acid, hexanoic acid, 3-methylbutanoic acid, decanoic acid), one furanone (2,5-dimethyl-4-hydroxy-2h-furan-3-one), two lactones (γ-decanolactone, 4-nonanolide), one sulfur-containing compound (3-methylthiopropanol), one aldehyde (3-methylbutanal), one ketone (3-hydroxy-2-butanone), and one other compound (myrcene).

To verify the specific contributions of these volatile compounds to the sensory quality of lager beer, both Pearson correlation analysis and flavor addition experiments were conducted. Pearson correlation analysis was used to evaluate the relationships between the OAV values of flavor compounds and the sensory scores of beer, thereby providing mathematical evidence of their influence on flavor expression. The flavor addition experiments involved spiking the selected compounds, grouped by chemical category, into the base beer, followed by sensory evaluation using the SQDA method.

**Figure 5:**
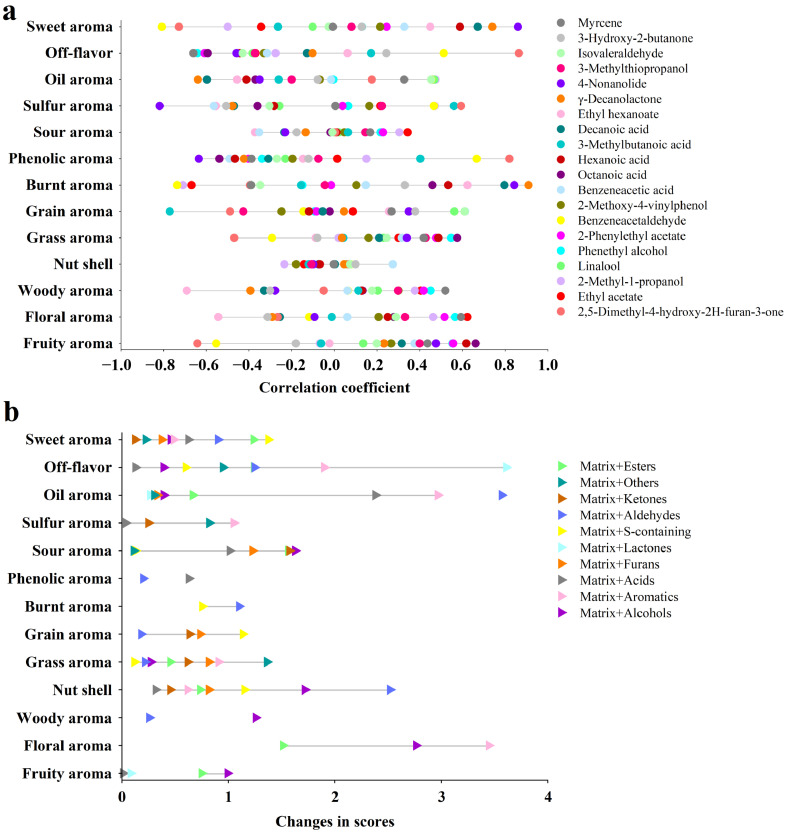
(**a**) Pearson correlation analysis between flavor compounds and sensory attributes. (**b**) Results of the flavor addition experiment.

The Pearson correlation results between the 20 flavor compounds and 13 aroma attributes of lager beer confirmed their distinct roles in flavor expression (Figure 5a). In the ester group, ethyl hexanoate was positively correlated with burnt aroma (r = 0.623), but moderately negatively correlated with woody aroma and floral aroma, suggesting enhancement of burnt characteristics while diminishing floral and woody impressions. Ethyl acetate showed a significant positive correlation with floral aroma (r = 0.622) and a strong negative correlation with burnt aroma (r = −0.669), indicating a greater contribution to fresh floral characteristics rather than to burnt notes.

For alcohols, linalool exhibited a moderate positive correlation with grain aroma (r = 0.561) and oil aroma (r = 0.467), suggesting that beyond floral enhancement, it may synergize with grain- and lipid-associated volatiles to enhance maltiness and smoothness [43,44].

Among aromatic compounds, phenethyl alcohol was positively correlated with fruity aroma (r = 0.553) and floral aroma (r = 0.564), and strongly negatively correlated with off-flavor (r = −0.642), indicating its role in elevating elegant aromatic notes while suppressing undesirable ones. 2-phenylethyl acetate showed similar correlations (r > 0.51 for fruity aroma and floral aroma; negative with off-flavor), highlighting the stable contribution of aromatic esters to desirable beer aroma profiles.

Within the acid group, octanoic acid was positively correlated with fruity aroma (r = 0.661) and sweet aroma (r = 0.588), and moderately negatively correlated with off-flavor, suggesting a role in imparting mild fruitiness and sweetness.

In the lactone group, γ-decanolactone exhibited extremely strong positive correlations with burnt aroma (r = 0.909) and sweet aroma (r = 0.739), while 4-nonanolide showed high correlations with burnt aroma (r = 0.842) and sweet aroma (r = 0.859), confirming the importance of lactones in sweet and baked-like aroma formation [45,46].

For furanones, 2,5-Dimethyl-4-hydroxy-2H-furan-3-one correlated strongly with phenolic (r = 0.819) and off-flavor (r = 0.864) attributes and negatively with sweet notes, suggesting that while it can add complexity, excessive levels may lead to undesirable flavors [47].

Overall, the Pearson correlation analysis revealed statistically significant relationships between specific volatiles and aroma attributes, indicating that these compounds shape the overall flavor profile of lager beer through both synergistic and antagonistic effects. It is important to clarify that while Pearson correlation analysis provides valuable information about statistical associations between chemical concentrations and sensory attributes, such correlations should not be misinterpreted as direct causal relationships. In this study, compounds showing significant correlations were subjected to additional validation through aroma recombination and flavor addition experiments. These experiments were designed to test the perceptual impact of specific volatiles under controlled conditions, thereby allowing us to move from correlation toward stronger evidence of causality.

The addition experiments (Figure 5b) further demonstrated that different types of volatile compounds contributed distinctly to beer aroma. Esters enhanced fruity, floral, sweet, acidic, and bready notes while reducing woody and phenolic characteristics, with Ethyl acetate and Ethyl hexanoate often exceeding their odor thresholds in beer [3,12]. Alcohols significantly increased floral, fruity, woody, bready, and acidic notes but decreased roasted, sulfur, and grain aromas, with linalool being a hop-derived odorant. Aromatic compounds enhanced floral, sweet, and oily notes, with 2-methoxy-4-vinylphenol identified as a key contributor to clove-like aroma [48]. Acids had limited effects on acidity but markedly increased oily notes and reduced grain aroma. Furanones reduced fruity and floral impressions. Lactones strongly enhanced sweetness, consistent with γ-decanolactone sensory results; notably, the BW sample showed the highest sweet score and lactone content. Sulfur compounds had minimal effects on sulfur notes but markedly increased off-flavor, possibly due to the sour and earthy odor of 3-methylthiopropanol [49]. Aldehydes enhanced oily and bready notes while suppressing fruitiness. Ketones mainly influenced acidic and sweet notes, whereas other compounds affected fruity and grassy characteristics.

In general, a single aroma attribute was influenced by multiple flavor compounds. Floral notes were primarily shaped by esters, alcohols, and aromatic compounds; sweet notes by esters, aromatics, lactones, and aldehydes; bready notes by alcohols, lactones, and aldehydes; and fruity notes by esters and alcohols. Previous studies have reported that fruity characteristics in beer are associated with high ester and relatively low alcohol concentrations [50]. 3-Methylbutanal, ethyl acetate, and benzeneacetaldehyde have been identified as aroma-active compounds in brewing barley [43], and 2,5-dimethyl-4-hydroxy-2H-furan-3-one has been repeatedly confirmed as aroma-active in caramel malt beer and wheat beer [18,20]. Collectively, the 20 flavor compounds were verified as key differential flavor compounds contributing to the aroma expression of different lager beer brands.

However, it should be noted that although the base matrix used in the addition experiment was a real beer (Yanjing Qing Shuang Beer), matrix effects may still influence the volatility, solubility, and perceptual intensity of the added compounds. In addition, the perception of aroma may also be modulated by cross-modal interactions, where taste attributes like bitterness or sweetness can influence how aromas are perceived. Although our study focused primarily on aroma-active volatiles, future investigations should incorporate more integrated designs to explore these multidimensional sensory interactions and their influence on flavor perception.

## 4. Conclusions

In this study, multiple sample pretreatment techniques were combined with advanced analytical instrumentation to comprehensively characterize the volatile compound profiles of four commercial lager beers. By integrating GC-MS, GC×GC-TOF-MS, and GC-O-MS with OAV/TAV calculation, multivariate statistical modeling, and sensory validation, we identified twenty differential volatile compounds as key contributors to brand-specific aroma expression. These compounds, predominantly aromatic compounds and acids, were found to shape typical sensory notes such as fruity, floral, caramel, and roasted characteristics through synergistic interactions. The integrated chemical-sensory approach adopted here offers a practical framework for exploring aroma complexity in fermented beverages and supports the development of flavor-targeted strategies in beer production. Despite these contributions, several limitations should be considered when interpreting the findings. The limited sample set, while representative of mainstream lager products in the Chinese market, may not capture the full diversity of lager styles across broader geographic or stylistic categories. Additionally, although storage conditions were controlled to minimize compound degradation, some volatile compounds remain highly sensitive to oxidation and evaporation, potentially influencing results. The detection of ultra-trace or unstable compounds also presents analytical challenges, even when employing advanced platforms. Building on these insights, future research should aim to address these limitations while deepening the understanding of aroma mechanisms. In particular, investigating the perceptual interactions between sub-threshold and supra-threshold aroma compounds may help clarify the sensory differences observed between recombination models and original beers.

## Data Availability

Data will be made available on request.

## Abbreviations

The following abbreviations are used in this manuscript:

LLE	Liquid–liquid extraction
HS-SPME	Headspace solid-phase microextraction
SAFE	Solvent-assisted flavor evaporation
GC-MS	Gas chromatography–mass spectrometry
GC×GC-TOF MS	Comprehensive two-dimensional gas chromatography–time-of-flight mass spectrometry
GC-O-MS	Gas chromatography–olfactometry–mass spectrometry
OAV	Odor activity value
TAV	Taste activity value
PLS-DA	Partial least squares discriminant analysis
VIP	Variable importance in projection
YJ	U8, Beijing Yanjing Brewery Co., Ltd.
QD	Classic, Tsingtao Brewery Group Co., Ltd.
HR	Brave the World, China Resources Beer (Holdings) Co., Ltd.
BW	Ice Beer, Budweiser Asia Pacific Holdings Ltd.

## Appendix A

**Table A1:** Brand, manufacturer, original wort concentration, and ingredients of lager beer samples used in this study.

Code	Product Name	Manufacturer	Original Wort Concentration	Ingredients
YJ	U8	Beijing Yanjing Brewery Co., Ltd.	8 °P	Water, malt, rice, hops
QD	Classic	Tsingtao Brewery Group Co., Ltd.	8 °P	Water, malt, rice, hops
HR	Brave the World	China Resources Beer (Holdings) Co., Ltd.	8 °P	Water, malt, brewing syrup, hops
BW	Ice Beer	Budweiser Asia Pacific Holdings Ltd.	8 °P	Water, malt, rice, hop extract, yeast
–	Qing Shuang Beer	Beijing Yanjing Brewery Co., Ltd.	10 °P	Water, malt, rice, hops

**Table A2:** Quantitative calibration curves of volatile compounds.

No.	Compound Name	CAS	Quantifier Ion	Calibration Curve				
				*n*	Slope	Intercept	R^2^	Range (mg/L)
1	Isoamyl acetate	123-92-2	43	9	0.00005	−15.2830	0.9972	2.34–1200.00
2	Ethyl hexanoate	123-66-0	88	8	0.000003	−0.9841	0.9993	6.25–800.00
3	Ethyl propanoate	105-37-3	29	7	0.0000005	1.2961	0.9998	0.31–160.00
4	Hexyl butanoate	2639-63-6	43	9	0.000003	3.6897	0.9974	1.25–320.00
5	Methyl decanoate	110-42-9	74	10	0.000003	0.8804	0.9990	0.78–400.00
6	Ethyl octanoate	106-32-1	88	7	0.0000002	−0.4058	0.9976	0.43–27.50
7	Ethyl decanoate	110-38-3	88	8	0.0000004	−0.1858	0.9997	0.16–40.00
8	Isoamyl butyrate	106-27-4	71	7	0.000004	−3.6818	0.9855	3.13–200.00
9	Ethyl trans-2-decenoate	7367-88-6	55	11	0.0000005	0.4049	0.9996	0.16–160.00
10	Octyl acetate	112-14-1	43	9	0.0000007	−0.3902	0.9998	0.39–100.00
11	Ethyl stearate	111-61-5	88	9	0.0000004	−1.2460	0.9994	0.39–100.00
12	Ethyl isovalerate	108-64-5	88	6	0.000001	−0.6628	0.9988	0.39–50.00
13	Ethyl myristate	124-06-1	88	7	0.0000004	−0.1408	0.9999	0.39–25.00
14	Ethyl lactate	97-64-3	45	7	0.000002	−0.6235	0.9977	0.78–50.00
15	Ethyl acetate	141-78-6	43	7	0.000003	−1.2344	0.9993	12.5–800.00
16	Ethyl heptanoate	106-30-9	88	8	0.0000008	−0.1803	0.9992	0.16–20.00
17	Hexyl acetate	142-92-7	43	8	0.000003	1.0401	0.9955	1.25–160.00
18	Diethyl succinate	123-25-1	101	10	0.0000009	0.1174	0.9995	0.31–160.00
19	Butyl acetate	123-86-4	43	9	0.000002	0.6073	0.9928	0.08–20.00
20	Ethyl 3-hydrobutyrate	5405-41-4	43	7	0.000002	0.0074	0.9997	0.02–1.25
21	Ethyl nonanoate	123-29-5	88	8	0.0000007	0.2045	0.9993	0.31–40.00
22	Ethyl hydrogen succinate	1070-34-4	101	6	0.000008	−1.0704	0.9992	10.00–320.00
23	Ethyl valerate	539-82-2	29	8	0.000002	0.3861	0.9909	0.31–40.00
24	Methyl 2-octynate	111-12-6	95	9	0.000003	−0.5746	0.9981	0.78–200.00
25	(−)-Ethyl L-lactate	687-47-8	45	8	0.000003	−1.9181	0.9973	0.78–100.00
26	2-Hydroxyethyl acetate	542-59-6	43	8	0.000002	0.1359	0.9926	0.16–20.00
27	Propyl acetate	109-60-4	43	8	0.000003	−1.9656	0.9971	0.16–20.00
28	2,3-Dihydroxypropyl acetate	106-61-6	43	9	0.000003	−1.7321	0.9967	0.16–40.00
29	Methyl propionate	554-12-1	29	9	0.000002	0.2601	0.9901	0.16–40.00
30	Isopropyl formate	625-55-8	45	8	0.000002	0.3989	0.9995	1.25–160.00
31	Ethyl dodecanoate	106-33-2	88	8	0.000002	0.6648	0.9987	0.16–20.00
32	Ethyl 3-hexenoate	2396-83-0	29	8	0.000002	1.7706	0.9918	0.31–40.00
33	Ethyl butyrylacetate	3249-68-1	71	8	0.000003	0.7792	0.9963	0.16–40.00
34	Ethyl 3-methylvalerate	5870-68-8	88	8	0.000001	−0.4345	0.9988	0.16–20.00
35	Ethyl undecanoate	627-90-7	88	8	0.000001	−1.6635	0.9984	0.16–20.00
36	(+)-Diethyl L-tartrate	87-91-2	104	8	0.000002	1.1348	0.9984	0.16–20.00
37	2,3-Butaneiol	513-85-9	45	9	0.00003	16.4440	0.9951	4.69–1200.00
38	3-Methyl-1-butanol	123-51-3	55	7	0.000006	−32.0320	0.9990	25.00–1600.00
39	1,2,3-Propanetriol	56-81-5	61	7	0.000004	124.5400	0.9992	125.00–8000.00
40	2-Methyl-1-propanol	78-83-1	43	9	0.00001	−6.9598	0.9996	4.69–1200.00
41	Diisobutylcarbinol	108-82-7	69	8	0.0000007	0.2155	0.9941	0.39–50.00
42	1-Butanol	71-36-3	56	8	0.000005	−0.6615	0.9998	1.56–200.00
43	1-Octanol	111-87-5	56	7	0.0000008	−0.1317	0.9995	0.31–20.00
44	1-Pentanol	71-41-0	42	10	0.000001	−0.4668	0.9991	0.31–320.00
45	3-Methyl-3-buten-1-ol	763-32-6	41	9	0.000006	0.0446	0.9966	0.04–10.00
46	α-Terpineol	98-55-5	59	6	0.0000007	0.0775	0.9993	0.16–5.00
47	2-Ethyl-1-hexanol	104-76-7	57	8	0.0000009	−0.6655	0.998	0.31–40.00
48	4-Methyl-1-pentanol	626-89-1	56	8	0.000003	0.7926	0.996	0.31–80.00
49	1,2-Propylene glycol	57-55-6	45	6	0.000005	1.6696	0.9993	1.25–40.00
50	2-Hexyl-1-decanol	2425-77-6	57	8	0.0000009	−0.6035	0.961	0.31–40.00
51	1-Hexanol	111-27-3	56	7	0.000004	−0.3672	0.9996	1.56–100.00
52	Linalool	78-70-6	71	8	0.000005	−0.5625	0.9998	0.78–100.00
53	1-Decanol	112-30-1	70	8	0.000005	−0.5974	0.9992	0.78–100.00
54	Diethylene glycol monoethyl ether	111-90-0	45	8	0.000005	−1.2538	0.9999	1.56–200.00
55	(−)-Isolongifolol	1139-17-9	109	7	0.000004	−0.0935	0.9997	1.56–100.00
56	Phytol	150-86-7	71	7	0.000004	0.1171	0.9996	0.39–25.00
57	β-Eudesmol	473-15-4	59	8	0.000001	0.0652	0.9982	3.13–200.00
58	Guaiol	489-86-1	161	9	0.000002	−0.3251	0.9997	0.78–200.00
59	2,6-Dimethyl-5,7-octadien-2-ol	5986-38-9	93	7	0.000001	0.0317	0.9964	1.56–100.00
60	Phenethyl alcohol	60-12-8	91	9	0.000007	−97.3390	0.9851	7.81–4000.00
61	2-Phenylethyl acetate	103-45-7	104	10	0.000003	−8.9800	0.9958	0.78–800.00
62	Phenol	108-95-2	94	10	0.000001	−4.5197	0.9993	1.17–600.00
63	Benzeneacetaldehyde	122-78-1	91	7	0.00001	0.5234	0.9916	1.25–80.00
64	Benzyl alcohol	100-51-6	79	7	0.0000009	0.0445	0.9999	0.16–10.00
65	Ethyl benzoate	93-89-0	105	7	0.000002	−0.0393	0.9997	0.63–40.00
66	Benzaldehyde	100-52-7	77	6	0.000003	0.0024	0.9904	1.25–40.00
67	2,4-Di-tert-butylphenol	96-76-4	191	8	0.000001	2.3582	0.9989	1.56–200.00
68	2-Methoxy-4-vinylphenol	7786-61-0	135	7	0.000002	5.609	0.9955	3.13–200.00
69	2,6-Di-tert-butyl-4-methylphenol	128-37-0	205	9	0.0000004	0.4619	0.9998	2.50–640.00
70	Benzoic acid	65-85-0	105	6	0.000003	6.6738	0.9964	12.5–400.00
71	p-Hydroxyphenylethanol	501-94-0	107	9	0.000001	2.0534	0.9989	0.78–200.00
72	Benzeneacetic acid	103-82-2	91	6	0.000003	4.3926	0.9943	6.25–200.00
73	Resorcinol	108-46-3	110	6	0.000002	1.6271	0.9992	1.56–50.00
74	2,5-Dimethylphenol	95-87-4	122	7	0.000001	1.6717	0.9967	0.78–50.00
75	α-Methylstyrene	98-83-9	118	6	0.000003	0.3579	0.9924	1.25–40.00
76	1-(2-Hydroxy-5-methylphenyl)ethanone	1450-72-2	135	8	0.000002	0.1579	0.9973	0.08–10.00
77	(R)-(+)-1-Phenylethanol	1517-69-7	107	8	0.000005	0.2135	0.9957	0.08–10.00
78	Toluene	108-88-3	91	6	0.000004	−1.9747	0.9986	7.81–250.00
79	Octanoic acid	124-07-2	60	8	0.000005	45.9690	0.9888	6.25–1600.00
80	Hexanoic acid	142-62-1	60	8	0.000008	−2.3383	0.9990	4.69–600.00
81	Acetic acid	64-19-7	43	6	0.00001	1.6743	0.9987	12.5–800.00
82	3-Methylbutanoic acid	503-74-2	60	8	0.000007	−0.1555	0.9967	3.13–400.00
83	Decanoic acid	334-48-5	60	6	0.000003	11.6970	0.9989	12.5–400.00
84	Dodecanoic acid	143-07-7	60	9	0.000004	3.1751	0.9996	3.13–800.00
85	Elaidic acid	112-79-8	55	8	0.000003	20.8040	0.9958	3.13–800.00
86	DL-3-Methylvaleric acid	105-43-1	60	8	0.000001	1.7304	0.9997	1.56–200.00
87	Heptanoic acid	111-14-8	60	9	0.000002	3.7580	0.9988	1.56–400.00
88	Tridecanoic acid	638-53-9	73	6	0.000001	1.8894	0.9915	0.78–50.00
89	2-Methylvaleric acid	594-61-6	59	6	0.000001	1.1722	0.9987	1.56–50.00
90	Pentanoic acid	109-52-4	60	7	0.000004	5.7311	0.9998	10.00–640.00
91	Nonanoic acid	112-05-0	60	8	0.0000002	0.4131	0.9962	0.31–40.00
92	Palmitic acid	57-10-3	43	6	0.000001	10.45	0.9993	12.5–400.00
93	trans-3-Hexenoic acid	4219-24-3	41	6	0.00001	6.6252	0.9978	0.78–25.00
94	4-Methyl-2-pentenoic acid	10321-71-8	41	8	0.000001	1.5656	0.9991	0.39–50.00
95	Hendecanoic acid	112-37-8	60	7	0.000002	1.1722	0.9987	0.39–25.00
96	Crotonic acid	3724-65-0	86	7	0.000002	−1.0557	0.9953	0.39–25.00
97	2-Methyl-2-pentenoic acid	3142-72-1	41	7	0.000006	−2.8264	0.9958	4.69–300.00
98	Furfuryl alcohol	98-00-0	98	6	0.000007	1.2221	0.9956	1.56–100.00
99	2-Furancarboxylic acid	88-14-2	112	6	0.000004	1.7403	0.9987	5.00–160.00
100	2-Butylfuran	4466-24-4	81	6	0.000004	1.6517	0.9948	1.25–80.00
101	Furfural	98-01-1	96	6	0.000002	2.345	0.9974	3.13–100.00
102	2-Acetyl furan	1192-62-7	95	11	0.000005	0.6997	0.9985	0.08–80.00
103	Methyl 2-furoate	611-13-2	95	7	0.000003	0.9577	0.9986	1.25–80.00
104	5-Hydroxymethyl-2-furaldehyde	67-47-0	97	9	0.000005	−7.9761	0.9971	4.68–1200.00
105	2,5-Dimethyl-4-hydroxy-2H-furan-3-one	3658-77-3	43	7	0.000006	3.1029	0.9981	1.56–100.00
106	2(5H)-Furanone	497-23-4	55	8	0.000008	−0.334	0.9933	1.56–200.00
107	Furfuryl sulfide	13678-67-6	81	7	0.000002	−1.394	0.9958	3.13–200.00
108	2,2′-Difurfuryl ether	4437-22-3	81	8	0.000003	−1.8112	0.9959	1.56–200.00
109	(±)-3-Hydroxy-4-butanolide	5469-16-9	44	7	0.00001	19.2420	0.9969	1.17–1200.00
110	γ-Decanolactone	706-14-9	85	7	0.0000006	−0.1815	0.9998	0.39–25.00
111	γ-Butyrolactone	96-48-0	42	10	0.00001	−7.8209	0.998	1.56–800.00
112	4-Nonanolide	104-61-0	85	6	0.000003	0.167	0.9991	0.78–50.00
113	γ-Hexanolactone	695-06-7	85	8	0.000001	0.0428	0.9999	0.08–10.00
114	γ-Octalactone	104-50-7	85	6	0.000004	−0.6705	0.9959	1.25–40.00
115	δ-Valerolactone	542-28-9	42	8	0.000001	0.1065	0.9992	0.08–10.00
116	γ-Valerolactone	108-29-2	56	10	0.000005	0.7945	0.9985	0.16–80.00
117	Costunolide	553-21-9	81	8	0.0000007	−0.4606	0.9992	0.39–50.00
118	5-Hydroxy-4-pentanolide	32780-06-6	85	6	0.000004	−0.3032	0.9977	2.50–80.00
119	2-Hydroxypyridine	142-08-5	95	7	0.000002	0.6471	0.9958	0.31–20.00
120	(Z)-9-Octadecenamide	301-02-0	59	6	0.0000007	0.0624	0.9963	2.50–80.00
121	2-Acetyl pyrrole	1072-83-9	94	7	0.000002	0.5324	0.9922	0.31–20.00
122	N-Butyl-acetamide	1119-49-9	30	8	0.000004	0.6875	0.9995	0.16–20.00
123	2,6-Di-tert-butylpyridine	585-48-8	176	9	0.000001	0.4103	0.994	0.16–40.00
124	5-Methyl-1H-pyrrole-2-carbaldehyde	1192-79-6	108	8	0.0000003	0.1641	0.9996	0.08–10.00
125	6-(Ethoxycarbonyl)nicotinic acid	17874-78-1	123	7	0.000001	−1.0354	0.9992	1.25–160.00
126	Benzylamine	100-46-9	106	7	0.000002	0.195	0.9981	0.63–40.00
127	Diethylamine	109-89-7	58	8	0.000003	0.2945	0.9963	0.08–10.00
128	3-Methylthiopropanol	505-10-2	106	10	0.000006	−1.3947	0.9995	1.56–800.00
129	Thiophene	110-02-1	84	9	0.0000005	−1.7580	0.9902	0.31–160.00
130	Methyl mercaptan	74-93-1	47	9	0.000006	−0.711	0.9982	1.56–100.00
131	Dimethyl Sulfoxide	67-68-5	63	8	0.00002	7.776	0.9962	3.13–400.00
132	Dimethyl sulfone	67-71-0	79	8	0.000007	0.5096	0.9975	0.31–40.00
133	Thioacetic acid	507-09-5	43	7	0.00002	9.9358	0.9964	3.13–200.00
134	Tetrahydrothiophene	110-01-0	60	7	0.00001	6.1179	0.995	1.56–100.00
135	2-Acetyl-2-thiazoline	29926-41-8	43	7	0.00002	−0.398	0.9984	6.25–400.00
136	4-Methyl-5-acetyl thiazole	38205-55-9	126	7	0.00003	0.7128	0.9961	1.56–100.00
137	(E)-2-Nonenal	18829-56-6	43	7	0.000001	0.4996	0.9996	1.56–100.00
138	Isovaleraldehyde	590-86-3	44	9	0.000001	−1.3097	0.9990	1.56–400.00
139	Acetaldehyde	75-07-0	29	8	0.000002	0.1996	0.9996	1.56–50.00
140	Hexadecanal	629-80-1	82	7	0.000001	0.2471	0.9991	0.78–50.00
141	4-Hydroxy-2-butanone	590–90-9	43	6	0.000002	3.5000	0.9997	5.00–160.00
142	(Z)-β-Ionone	79-77–6	177	8	0.000002	2.9139	0.9992	1.56–200.00
143	3-Hydroxy-2-butanone	513-86–0	45	8	0.000002	1.3398	0.9987	0.63–160.00
144	Cholestane	481-21-0	217	8	0.000003	1.1282	0.9989	3.13–400.00
145	1-Heptene	592-76-7	56	7	0.00003	−10.3550	0.9955	0.39–50.00
146	1-Undecene	821-95-4	43	9	0.0000008	−0.5146	0.9998	0.16–80.00
147	2-Methyl-2-butene	513-35-9	55	7	0.000002	1.0146	0.9972	0.78–50.00
148	Myrcene	123-35-3	41	7	0.000002	1.0361	0.9986	0.78–50.00
149	Diisopropyl ether	108-20-3	45	7	0.000002	1.8727	0.9972	0.78–50.00
150	3-Methyl-1-butene	563-45-1	55	7	0.000003	0.0584	0.9993	0.78–50.00

**Table A3:** Identification of volatile compounds in different brands of lager beer.

No.	Compound Name	CAS	RI		Identification Method	Sample Preparation	Presence in Samples			
			DB-WAX	DB-FFAP			YJ	QD	HR	BW
Alcohols (98)										
1	2-Propen-1-ol	107-18-6	1016		MS, RI	LLE-SAFE	√	√	√	√
2	1-Methoxy-2-propanol	107-98-2	1028		MS, RI	LLE-SAFE	√	√	√	√
3	1-Butanol	71-36-3	1044		MS, RI, S	LLE-SAFE	√	√	√	√
4	3-Penten-2-ol	1569-50-2	1070		MS, RI	LLE-SAFE	√	√	√	√
5	(±)-2-Methyl-1-butanol	137-32-6	1104	1145	MS, RI, S	LLE-SAFE, SPME	√	√	√	√
6	(S)-(−)-2-Methyl-1-butanol	1565-80-6	1106	1141	MS, RI	LLE-SAFE, SPME	√	√	√	√
7	3-Methyl-1-butanol	123-51-3	1107	1131	MS, RI, aroma, S	LLE-SAFE, SPME	√	√	√	√
8	3-Butyn-2-ol	2028-63-9	1111		MS, RI	LLE-SAFE	√			
9	3-Methyl-3-buten-1-ol	763-32-6	1147		MS, RI, S	LLE-SAFE	√	√	√	√
10	1-Pentanol	71-41-0	1149		MS, RI, S	LLE-SAFE	√	√	√	√
11	2-Cyclopropylethanol	2566-44-1	1200		MS, RI	LLE-SAFE	√	√	√	√
12	4-Penten-1-ol	821-09-0	1200		MS, RI	LLE-SAFE	√	√	√	√
13	4-Methyl-1-pentanol	626-89-1	1213	1205	MS, RI, S	LLE-SAFE, SPME	√			√
14	2-Methyl-2-buten-1-ol	4675-87-0	1219		MS, RI	LLE-SAFE			√	
15	3-Methyl-2-buten-1-ol	556-82-1	1219		MS, RI	LLE-SAFE	√	√	√	√
16	2-Pentyn-1-ol	6261-22-9	1235		MS, RI	LLE-SAFE		√		
17	Cyclobut-1-enylmethanol	89182-08-1	1235		MS, RI	LLE-SAFE	√	√	√	√
18	1,4-Pentadien-3-ol	922-65-6	1235		MS, RI	LLE-SAFE		√		
19	2-Methoxyethanol	109-86-4	1236		MS, RI	LLE-SAFE			√	√
20	1-Hexanol	111-27-3	1252	1227	MS, RI, S	LLE-SAFE, SPME	√	√	√	√
21	4-Methyl-3-penten-1-ol	763-89-3	1285		MS, RI	LLE-SAFE				√
22	Propargyl alcohol	107-19-7	1294		MS, RI	LLE-SAFE				√
23	1-Heptanol	111-70-6	1352		MS, RI, S	LLE-SAFE	√			
24	(2S)-2-Oxiranylmethanol	60456-23-7	1356		MS, RI	LLE-SAFE	√		√	
25	2-Ethyl-1-hexanol	104-76-7	1387	1311	MS, RI, S	LLE-SAFE, SPME				√
26	2-Methylbutane-2,3-diol	5396-58-7	1411		MS, RI	LLE-SAFE			√	√
27	(R)-(−)-2-Hexanol	26549-24-6	1418		MS, RI	LLE-SAFE	√			
28	(R)-2-Octanol	5978-70-1	1418		MS, RI	LLE-SAFE	√			
29	(2R,3R)-(−)-2,3-Butanediol	513-85-9	1434	1541	MS, RI, aroma, S	LLE-SAFE, SPME	√	√	√	√
30	(S,S)-(+)-1,3-Butaneiol	19132-06-0	1435		MS, RI	LLE-SAFE	√	√		√
31	Linalool	78-70-6	1443	1332	MS, RI	LLE-SAFE, SPME	√	√	√	
32	1-Octanol	111-87-5	1456	1348	MS, RI, S	LLE-SAFE, SPME	√	√	√	√
33	2-(2-Methoxyethoxy)ethanol	111-77-3	1480	1955	MS, RI	LLE-SAFE, SPME				√
34	(S)-(+)-1,2-Propanediol	4254-15-3	1484		MS, RI	LLE-SAFE	√	√	√	√
35	1,2-Propylene glycol	57-55-6	1484	1592	MS, RI, aroma, S	LLE-SAFE	√	√	√	√
36	3-Ethoxy-1-propanol	111-35-3	1507		MS, RI	LLE-SAFE	√		√	√
37	Ethylene glycol	107-21-1	1516	1627	MS, RI	LLE-SAFE	√	√	√	√
38	Cyclopropanemethanol	2516-33-8	1545		MS, RI	LLE-SAFE	√	√	√	√
39	Cyclobutanol	2919-23-5	1545		MS, RI	LLE-SAFE	√			√
40	1-Methoxy-2-butanol	53778-73-7	1550		MS, RI	LLE-SAFE	√	√	√	√
41	1,2-Butanediol	584-03-2	1550		MS, RI	LLE-SAFE		√		
42	α-Terpineol	98-55-5	1593	1386	MS, RI, S	LLE-SAFE, SPME	√	√	√	
43	4,5-Octanediol	22607-10-9	1598		MS, RI	LLE-SAFE			√	
44	2-Nonanol	628-99-9	1618		MS, RI	LLE-SAFE	√			
45	1,3-Butaneiol	107-88-0	1634		MS, RI	LLE-SAFE		√	√	√
46	2-Methyl-3-hexanol	617-29-8	1639		MS, RI	LLE-SAFE			√	
47	1-Decanol	112-30-1	1659	1420	MS, RI	LLE-SAFE, SPME		√	√	√
48	1-Nonanol	143-08-8	1659		MS, RI, S	LLE-SAFE	√	√		√
49	5-Methyl-1-hepten-4-ol	99328-46-8	1676		MS, RI	LLE-SAFE		√		
50	2-(2-Butoxyethoxy)ethanol	112-34-5	1688		MS, RI	LLE-SAFE		√		
51	2,2-Dimethyl-1,3-propanediol	126-30-7	1697		MS, RI	LLE-SAFE	√			
52	1,1-Oxydi-2-propanol	110-98-5	1726	2389	MS, RI	LLE-SAFE, SPME		√	√	√
53	2-(2-Hydroxypropoxy)-1-propanol	106-62-7	1777		MS, RI	LLE-SAFE		√		
54	Diethylene glycol	111-46-6	1865		MS, RI	LLE-SAFE	√	√	√	√
55	1-Nonene-4-ol	35192-73-5	1886		MS, RI	LLE-SAFE	√	√	√	√
56	1,2-Heptanediol	3710-31-4	1886		MS, RI	LLE-SAFE	√		√	√
57	1,6-Heptadien-4-ol	2883-45-6	1978		MS, RI	LLE-SAFE	√	√		√
58	1-Buten-3-ol	598-32-3	1997		MS, RI	LLE-SAFE	√		√	
59	2-Methyl-1-penten-3-ol	2088/7/5	2012		MS, RI	LLE-SAFE		√		
60	2-Hexen-4-ol	4798-58-7	2012		MS, RI	LLE-SAFE				√
61	1,2,3-Propanetriol	56-81-5	2197	2323	MS, RI, S	LLE-SAFE	√	√	√	√
62	Triethylene glycol	112-27-6	2216	1942	MS, RI	LLE-SAFE, SPME	√	√	√	√
63	2-Methyl-2-propanol	75-65-0	2219		MS, RI, S	LLE-SAFE			√	
64	2E,6E-Farnesol	106-28-5	2249		MS, RI, S	LLE-SAFE		√	√	
65	Farnesol	4602-84-0	2249		MS, RI	LLE-SAFE		√		
66	2,2-Dimethyl-5-hexen-3-ol	19550-89-1	2261		MS, RI	LLE-SAFE			√	
67	3-Decyn-2-ol	69668-93-5	2469		MS, RI	LLE-SAFE				√
68	2,3-Epoxy-1-propanol	57044-25-4	2476		MS, RI	LLE-SAFE	√		√	√
69	1-Dodecanol	112-53-8	2481		MS, RI	LLE-SAFE				√
70	3-Methyl-2-hexanol	2313-65-7	2503		MS, RI	LLE-SAFE	√			
71	2-(Vinyloxy)ethanol	764-48-7	2503	1685	MS, RI	LLE-SAFE, SPME	√	√	√	√
72	2-Butene-1,4-diol	110-64-5	2561		MS, RI	LLE-SAFE				√
73	Isopropyl alcohol	67-63-0	2596		MS, RI, S	LLE-SAFE				√
74	Di(ethylene glycol) vinyl ether	929-37-3	2600		MS, RI	LLE-SAFE	√			√
75	(R)-(−)-1,2-Propanediol	4254-14-2	2816		MS, RI	LLE-SAFE	√		√	
76	4-Penten-2-ol	625-31-0	2946		MS, RI	LLE-SAFE				√
77	2-Eyhoxyethanol	110-80-5	3099		MS, RI	LLE-SAFE			√	√
78	Glycidol	556-52-5	3220		MS, RI	LLE-SAFE	√	√	√	√
79	2-Methyl-1-propanol	78-83-1	996	682	MS, RI, S	LLE-SAFE, SPME	√	√	√	√
80	4-Methyl-2-pentanol	108-11-2		2241	MS, RI	SPME	√	√		
81	Diisobutylcarbinol	108-82-7		1215	MS, RI, S	SPME	√		√	√
82	2,5-Dimethyl-2,5-hexanediol	110-03-2		1269	MS, RI	SPME				√
83	(+)-β-Citronellol	1117-61-9		1340	MS, RI	SPME			√	
84	Diethylene glycol monoethyl ether	111-90-0		2248	MS, RI	SPME	√	√	√	√
85	(−)-Isolongifolol	1139-17-9		2421	MS, RI	LLE-SAFE		√		√
86	Phytol	150-86-7		1061	MS, RI	SPME	√	√	√	
87	2-Methyl-3-heptanol	18720-62-2		1217	MS, RI	SPME		√	√	√
88	4-Methyl-4-nonanol	23418-38-4		2402	MS, RI	SPME	√			√
89	2-Hexyl-1-decanol	2425-77-6		2377	MS, RI, aroma, S	LLE-SAFE	√		√	
90	β-Eudesmol	473-15-4			MS, S	LLE-SAFE	√	√	√	√
91	Pentaethylene glycol	4792-15-8		2006	MS, RI	SPME	√	√	√	√
92	Guaiol	489-86-1		2203	MS, RI, S	SPME		√	√	√
93	Octaethylene glycol	5117-19-1		1986	MS, RI	SPME	√	√	√	√
94	2,2,4-Trimethyl-3-pentanol	5162-48-1		1261	MS, RI	SPME		√		√
95	Heptaethylene glycol	5617-32-3		2082	MS, RI	SPME	√	√	√	√
96	1-Methylcyclohexanol	590-67-0		1050	MS, RI	SPME	√		√	√
97	2,6-Dimethyl-5,7-octadien-2-ol	5986-38-9		1387	MS, RI	SPME	√		√	
98	5-Nonanol	623-93-8		1273	MS, RI	SPME			√	
Nitrogen-containing compounds (91)										
1	Triethylamine	121-44-8	1110		MS, RI	LLE-SAFE		√		
2	N,N,O-Triacetylhydroxylamine	17720-63-7	1112		MS, RI	LLE-SAFE		√	√	√
3	2-Methylpyrazine	109-08-0	1162		MS, RI, S	LLE-SAFE	√		√	√
4	N,N-Dimethylformamide	1968-12-2	1223		MS, RI	LLE-SAFE	√	√	√	√
5	2-Oxopropanamide	631-66-3	1531		MS, RI	LLE-SAFE		√		√
6	N-Methylacetamide	79-16-3	1533		MS, RI	LLE-SAFE				√
7	N-Ethylacetamide	625-50-3	1533		MS, RI	LLE-SAFE	√	√	√	√
8	N-Acetyglycinamide	2620-63-5	1610		MS, RI	LLE-SAFE			√	
9	2,3-Butanedione monoxime	57-71-6	1627		MS, RI	LLE-SAFE	√		√	
10	Acetamide	60-35-5	1656		MS, RI	LLE-SAFE	√		√	√
11	Formamide	1975/12/7	1675		MS, RI	LLE-SAFE	√	√	√	√
12	3-(Ethoxycarbonyl)pyridine	614-18-6	1706		MS, RI	LLE-SAFE	√	√	√	√
13	N-Methylsuccinimide	1121-07-9	1793		MS, RI	LLE-SAFE	√		√	√
14	2-Pyridinecarbaldehyde oxime	873-69-8	1806		MS, RI	LLE-SAFE			√	
15	3-Methylbutyramide	541-46-8	1811		MS, RI	LLE-SAFE	√			√
16	2-Acetyl pyrrole	1072-83-9	1862	1978	MS, RI, aroma, S	LLE-SAFE	√	√	√	√
17	3-Benzylsydnone	16844-42-1	1862		MS, RI	LLE-SAFE			√	
18	2-Pyrrolecarbaldehyde	1003-29-8	1895		MS, RI	LLE-SAFE	√	√	√	√
19	2-Pyrrolidinone	616-45-5	1928		MS, RI	LLE-SAFE	√	√	√	√
20	1-Methylpyrrole-2-carboxaldehyde	1192-58-1	1989		MS, RI	LLE-SAFE	√	√	√	√
21	Butanamide	541-35-5	2000		MS, RI	LLE-SAFE	√	√	√	√
22	Hexanamide	628-02-4	2000		MS, RI	LLE-SAFE	√		√	√
23	5-Aminopentanamide	13023-70-6	2021		MS, RI	LLE-SAFE			√	
24	2-Piperidinone	675-20-7	2021		MS, RI	LLE-SAFE				√
25	N-Acetylglycine ethyl ester	1906-82-7	2044		MS, RI	LLE-SAFE	√	√	√	√
26	ε-Caprolactam	105-60-2	2074		MS, RI	LLE-SAFE	√	√	√	√
27	3,5-Dihydroxy-6-methyl-2,3-dihydro-4H-pyran-4-one	28564-83-2	2157		MS, RI	LLE-SAFE	√	√	√	√
28	Ethosuximide	77-67-8	2252		MS, RI	LLE-SAFE	√	√	√	√
29	Glutarimide	1121-89-7	2264		MS, RI	LLE-SAFE	√		√	√
30	N,N,3,5-Tetramethylaniline	4913-13-7	2269		MS, RI	LLE-SAFE	√			
31	N-Acetylethanolamine	142-26-7	2277		MS, RI	LLE-SAFE		√	√	
32	Indole	120-72-9	2329		MS, RI	LLE-SAFE	√	√	√	√
33	5H-cyclopenta[b]pyridine	270-91-7	2329		MS, RI	LLE-SAFE	√	√	√	
34	Succinimide	123-56-8	2355		MS, RI	LLE-SAFE				√
35	N,S-Diacetylcysteamine	1420-88-8	2411		MS, RI	LLE-SAFE	√	√	√	
36	N-(2-Phenylethyl)acetamide	877-95-2	2473		MS, RI	LLE-SAFE		√		
37	1-Methyl-1H-tetrazole	16681-77-9	2475		MS, RI	LLE-SAFE				√
38	4-Amino-1-butanol	13325-10-5	2479		MS, RI	LLE-SAFE	√			
39	DL-Pyroglutamic acid	149-87-1	2497		MS, RI	LLE-SAFE		√		
40	Ethyl 5-oxo-L-prolinate	66183-71-9	2497		MS, RI	LLE-SAFE	√	√	√	√
41	L-Pyroglutamicacid	98-79-3	2500		MS, RI	LLE-SAFE	√			
42	Benzamide	55-21-0	2575		MS, RI	LLE-SAFE		√		√
43	Phenylacetamide	103-81-1	2607		MS, RI	LLE-SAFE			√	
44	Valeramide	626-97-1	2654		MS, RI	LLE-SAFE		√	√	√
45	Enanthamide	628-62-6	2657		MS, RI	LLE-SAFE	√			
46	Octanamide	629-01-6	2657		MS, RI	LLE-SAFE	√	√	√	√
47	Pent-4-enylamine	22537-07-1	2705		MS, RI	LLE-SAFE		√		
48	Cyclobutylamine	2516-34-9	2762		MS, RI	LLE-SAFE	√		√	√
49	3-Indoleethanol	526-55-6	2767		MS, RI	LLE-SAFE		√		
50	(2-Hydroxyethyl)hydrazine	109-84-2	2811		MS, RI	LLE-SAFE		√		
51	Dodecanamide	1120-16-7	2883		MS, RI	LLE-SAFE		√		√
52	(Z)-9-Octadecenamide	301-02-0	2887	2432	MS, RI, aroma, S	LLE-SAFE	√	√	√	√
53	2-Propoxyethylamine	42185-03-5	2937		MS, RI	LLE-SAFE			√	
54	2-Methoxyethylamine	109-85-3	2963		MS, RI	LLE-SAFE			√	√
55	2-Ethoxyethylamine	110-76-9	2968		MS, RI	LLE-SAFE				√
56	Nonanamide	1120-07-6	3105		MS, RI	LLE-SAFE	√	√	√	√
57	1,2-Propanediamine	78-90-0	3147		MS, RI	LLE-SAFE			√	√
58	N-Methylisobutylamine	625-43-4	3222		MS, RI	LLE-SAFE			√	
59	N-Methyltyramine	370-98-9	884		MS, RI	LLE-SAFE		√		√
60	4-Hydroxypyrazole	4843-98-5	924		MS, RI	LLE-SAFE	√			
61	4H-1,2,4-Triazol-4-amine	584-13-4	925		MS, RI	LLE-SAFE		√		
62	Benzylamine	100-46-9		1188	MS, RI	SPME	√			√
63	2,4,6-Triamino-5-nitrosopyrimidine	1006-23-1			MS	LLE-SAFE		√		
64	1,3,5-Trimethylpyrazole	1072-91-9		2157	MS, RI	LLE-SAFE		√	√	
65	Diethylamine	109-89-7		597	MS, RI	SPME	√			√
66	N-Butyl-acetamide	1119-49-9		1118	MS, RI, aroma, S	LLE-SAFE, SPME		√	√	√
67	cis-13-Docosenoamide	112-84-5			MS	LLE-SAFE			√	√
68	5-Methyl-1H-pyrrole-2-carbaldehyde	1192-79-6		2120	MS, RI, aroma, S	LLE-SAFE	√			√
69	Octadecanamide	124-26-5			MS	LLE-SAFE		√	√	
70	2-Hydroxypyridine	142-08-5		1468	MS, RI, aroma, S	LLE-SAFE			√	√
71	6-(Ethoxycarbonyl)nicotinic acid	17874-78-1			MS, aroma, S	LLE-SAFE			√	√
72	Diethyl iminodiacetate	19617-44-8		2035	MS, RI	SPME	√	√	√	√
73	N-Ethylisopropylamine	19961-27-4		1270	MS, RI	SPME		√	√	√
74	2-Hydroxy-Propanamide	2043-43-8		667	MS, RI	SPME			√	√
75	5,6,7,8-Tetrahydro-1-naphthylamine	2217-41-6		2039	MS, RI	SPME		√		
76	4-Hydroxyindole	2380-94-1		2284	MS, RI	SPME			√	
77	5-Amino-1-pentanol	2508-29-4		1043	MS, RI	SPME			√	
78	Pyrazole	288-13-1		2243	MS, RI	LLE-SAFE	√			
79	4-Hydroxy-6-methylpyrimidine	3524-87-6		2172	MS, RI	LLE-SAFE			√	
80	2-Amino-1,3,4-thiadiazole	4005-51-0		1294	MS, RI	SPME	√			
81	N-Ethylmaleamic acid	4166-67-0		2233	MS, RI	SPME	√	√	√	√
82	N-Ethyl-pentanamide	54007-33-9		1267	MS, RI	SPME		√	√	√
83	Isobutyramide	563-83-7		590	MS, RI	SPME	√	√		
84	2,6-Di-tert-butylpyridine	585-48-8		2457	MS, RI, aroma, S	LLE-SAFE	√	√		√
85	Ethylhydrazine	624-80-6		1710	MS, RI	SPME			√	
86	Imidazole-4-acetic acid	645-65-8		1185	MS, RI	SPME	√	√		
87	3-Hydroxypiperidine	6859-99-0		1194	MS, RI	SPME		√		
88	N,N-Dimethylthioformamide	758-16-7		1786	MS, RI	SPME	√	√	√	√
89	N,N-Dimethylpropanamide	758-96-3		664	MS, RI	SPME			√	√
90	Ethyl 3-aminopropanoate	924-73-2		1269	MS, RI	SPME		√		
91	2-Ethyl-4-methylimidazole	931-36-2			MS	LLE-SAFE	√	√		
Esters (86)										
1	Isoamyl acetate	123-92-2	1024	1014	MS, RI, aroma, S	LLE-SAFE, SPME	√	√	√	√
2	2-Methylbutyl acetate	624-41-9	1024		MS, RI	LLE-SAFE	√		√	
3	Pentyl acetate	628-63-7	1024		MS, RI, S	LLE-SAFE		√		√
4	Ethyl valerate	539-82-2	1038	1433	MS, RI, S	LLE-SAFE	√	√	√	√
5	Ethyl hexanoate	123-66-0	1135	1157	MS, RI, aroma, S	LLE-SAFE, SPME	√	√	√	√
6	Ethyl Pyruvate	617-35-6	1164		MS, RI	LLE-SAFE	√	√	√	√
7	Hexyl acetate	142-92-7	1171	1183	MS, RI, S	LLE-SAFE, SPME	√	√	√	√
8	Allyl butanoate	2051-78-7	1175		MS, RI	LLE-SAFE	√	√	√	√
9	Ethyl heptanoate	106-30-9	1232	1214	MS, RI, S	LLE-SAFE, SPME	√	√	√	√
10	(−)-Ethyl L-lactate	687-47-8	1238		MS, RI	LLE-SAFE	√	√	√	√
11	Ethyl lactate	97-64-3	1238		MS, RI, S	LLE-SAFE	√	√	√	√
12	Hexyl formate	629-33-4	1250		MS, RI, S	LLE-SAFE				√
13	2-Propenoic acid ethenyl ester	2177-18-6	1294		MS, RI	LLE-SAFE	√	√		√
14	Methyl acrylate	96-33-3	1294		MS, RI	LLE-SAFE			√	√
15	Ethyl octanoate	106-32-1	1332	1303	MS, RI, S	LLE-SAFE, SPME	√	√	√	√
16	Heptyl formate	112-23-2	1352		MS, RI	LLE-SAFE	√			
17	3-Methoxybutyl acetate	4435-53-4	1411		MS, RI	LLE-SAFE		√		
18	Ethyl 2-hydroxyisobutyrate	80-55-7	1411		MS, RI	LLE-SAFE		√	√	
19	Ethyl 3-hydrobutyrate	5405-41-4	1411	1876	MS, RI, S	LLE-SAFE, SPME			√	√
20	Octyl formate	112-32-3	1454		MS, RI	LLE-SAFE		√	√	
21	2-Hydroxypropyl acetate	627-69-0	1467		MS, RI	LLE-SAFE	√			
22	Ethyl levulinate	539-88-8	1500		MS, RI	LLE-SAFE			√	
23	2-Hydroxyethyl formate	628-35-3	1514		MS, RI	LLE-SAFE	√	√	√	√
24	2-Hydroxyethyl acetate	542-59-6	1528		MS, RI	LLE-SAFE	√	√	√	√
25	Ethyl decanoate	110-38-3	1535	1377	MS, RI, S	LLE-SAFE, SPME	√		√	√
26	Diethyl succinate	123-25-1	1570		MS, RI, S	LLE-SAFE	√	√	√	√
27	Dimethyl glutarate	1119-40-0	1593		MS, RI	LLE-SAFE	√	√		
28	Isopropyl isobutyrate	617-50-5	1600		MS, RI	LLE-SAFE			√	
29	Propyl acetate	109-60-4	1630		MS, RI, S	LLE-SAFE	√	√		
30	1,3-Diacetoxy-propane	628-66-0	1632		MS, RI	LLE-SAFE	√	√	√	√
31	Neryl formate	2142-94-1	1744		MS, RI	LLE-SAFE	√			
32	1,1-Ethanediol diacetate	542-10-9	1749		MS, RI	LLE-SAFE			√	√
33	Butyl butyrate	109-21-7	1760		MS, RI	LLE-SAFE		√		
34	Butyl 2-methyl propanoate	97-87-0	1760		MS, RI, S	LLE-SAFE		√		
35	Pentyl 2-methylprop-2-enoate	2849-98-1	1936		MS, RI	LLE-SAFE				√
36	Allyl propionate	2408-20-0	2012		MS, RI	LLE-SAFE			√	
37	Vinyl butyrate	123-20-6	2012		MS, RI	LLE-SAFE		√		
38	Allyl isobutyrate	15727-77-2	2015		MS, RI	LLE-SAFE	√		√	
39	Ethyl cinnamate	103-36-6	2019		MS, RI	LLE-SAFE				√
40	Vinyl acetate	108-05-4	2038		MS, RI	LLE-SAFE	√			
41	Methyl acetate	79-20-9	2102		MS, RI, S	LLE-SAFE	√			
42	Methyl tridecanoate	1731-88-0	2112		MS, RI	LLE-SAFE		√		
43	Glycidyl acrylate	106-90-1	2139		MS, RI	LLE-SAFE			√	√
44	2,3-Dihydroxypropyl acetate	106-61-6	2173		MS, RI	LLE-SAFE	√	√	√	√
45	Ethyl cyclohexanepropionate	10094-36-7	2233		MS, RI	LLE-SAFE	√	√	√	√
46	Ethyl nonanoate	123-29-5	2233	1285	MS, RI, S	LLE-SAFE, SPME	√	√	√	√
47	Allyl propyl ether	1471-03-0	2589		MS, RI	LLE-SAFE	√			
48	2-Oxooctadecanoic acid methyl ester	2380-18-9	2607		MS, RI	LLE-SAFE	√			
49	L-Lactide	4511-42-6	2630		MS, RI	LLE-SAFE	√			
50	Ethenyl formate	692-45-5	2716		MS, RI	LLE-SAFE		√	√	
51	Methyl propionate	554-12-1	2729		MS, RI, S	LLE-SAFE, SPME	√	√		
52	Butyl lactate	138-22-7	2751		MS, RI, S	LLE-SAFE			√	√
53	Lactic acid methyl ester	547-64-8	2811		MS, RI	LLE-SAFE			√	
54	Isopropyl formate	625-55-8	2939		MS, RI	LLE-SAFE	√		√	
55	DL-Lactide	95-96-5	3127		MS, RI	LLE-SAFE			√	√
56	Ethyl acetate	141-78-6	913	606	MS, RI, S	LLE-SAFE, SPME	√	√	√	√
57	Isobutyl acetate	110-19-0	990		MS, RI, S	LLE-SAFE	√			
58	Butyl acetate	123-86-4	990		MS, RI, S	LLE-SAFE			√	√
59	2,2-Dimethoxypropionic acid methyl ester	10076-48-9		2237	MS, RI	SPME	√		√	
60	sec-Butyl Crotonate	10371-45-6		1304	MS, RI	SPME	√	√		
61	Ethyl propanoate	105-37-3		2101	MS, RI, aroma, S	LLE-SAFE	√	√	√	√
62	Isoamyl butyrate	106-27-4		1102	MS, RI, S	SPME	√			
63	Ethyl dodecanoate	106-33-2		2256	MS, RI	LLE-SAFE	√		√	
64	Ethyl hydrogen succinate	1070-34-4		2388	MS, RI, aroma, S	LLE-SAFE	√	√	√	√
65	Ethyl isovalerate	108-64-5		1302	MS, RI, S	SPME		√		
66	Methyl decanoate	110-42-9		2215	MS, RI, aroma, S	LLE-SAFE	√	√	√	√
67	Methyl 2-octynate	111-12-6		2415	MS, RI, aroma, S	LLE-SAFE			√	√
68	Ethyl Stearate	111-61-5		1301	MS, RI, S	SPME	√	√	√	√
69	Octyl Acetate	112-14-1		1298	MS, RI, S	SPME	√	√		
70	Ethyl myristate	124-06-1		1382	MS, RI, S	SPME	√	√	√	√
71	Ccrotonic acid isopropyl ester	18060-77-0		1263	MS, RI	SPME	√	√		√
72	Ethyl nonadecanoate	18281-04-4		1299	MS, RI	SPME	√			√
73	Methyl (Z)-3,7-dimethylocta-2,6-dienoate	1862-61-9		1394	MS, RI	SPME	√			
74	Ethyl 3-hexenoate	2396-83-0		1206	MS, RI, S	SPME	√	√		√
75	2-Ethylhexyl Butyrate	25415-84-3		1320	MS, RI	SPME		√		
76	Hexyl butanoate	2639-63-6		2144	MS, RI, aroma, S	LLE-SAFE	√	√	√	√
77	Ethyl butyrylacetate	3249-68-1		2297	MS, RI	SPME	√	√	√	
78	Heptadecanyl margarate	36617-50-2		1422	MS, RI	SPME		√		√
79	Dimethyl dl-malate	38115-87-6		1197	MS, RI	SPME		√	√	
80	Methyl 4-methoxyacetoacetate	41051-15-4		2275	MS, RI	SPME	√			
81	Ethyl 3-methylvalerate	5870-68-8		1299	MS, RI	SPME	√			
82	Ethyl undecanoate	627-90-7		1271	MS, RI	SPME	√		√	
83	Dodecyl isobutyrate	6624-71-1		2332	MS, RI	SPME		√	√	
84	Ethyl trans-2-decenoate	7367-88-6		1194	MS, RI, S	SPME			√	
85	Isobutyric acid 2-ethyl-3-hydroxyhexyl ester	74367-31-0		677	MS, RI	SPME	√			
86	(+)-Diethyl L-tartrate	87-91-2		1526	MS, RI	SPME			√	
Alkanes (57)										
1	1,1-Dimethyl cyclopropane	1630-94-0	886		MS, RI	LLE-SAFE	√	√	√	√
2	s-Trioxane	110-88-3	1063		MS, RI	LLE-SAFE	√	√	√	√
3	Nonane	111-84-2	1105		MS, RI	LLE-SAFE			√	
4	2,4-Dimethyldecane	2801-84-5	1105		MS, RI	LLE-SAFE			√	
5	n-Hendecane	1120-21-4	1105		MS, RI	LLE-SAFE			√	
6	2,3-Dimethylbutane	79-29-8	1111		MS, RI	LLE-SAFE	√			
7	Decane	124-18-5	1201		MS, RI	LLE-SAFE			√	√
8	3,7-Dimethyldecane	17312-54-8	1201		MS, RI	LLE-SAFE		√		
9	2-Methylnonane	871-83-0	1201		MS, RI	LLE-SAFE				√
10	Tridecane	629-50-5	1203		MS, RI	LLE-SAFE		√	√	√
11	Propyl cyclopropane	2415-72-7	1250		MS, RI	LLE-SAFE			√	
12	Cyclododecane	294-62-2	1659		MS, RI	LLE-SAFE	√			
13	2,3-Dimethyloxirane	3266-23-7	1713		MS, RI	LLE-SAFE				√
14	1,4- Diacetoxybutane	628-67-1	1769		MS, RI	LLE-SAFE		√		
15	trans-1,2-Dimethylcyclopropane	2402/6/4	2039		MS, RI	LLE-SAFE		√		
16	1,2-Epoxyhexane	1436-34-6	2155		MS, RI	LLE-SAFE			√	√
17	Methylcyclobutane	598-61-8	2264		MS, RI	LLE-SAFE	√		√	
18	6-Ethyl-2-methyldecane	62108-21-8	2401		MS, RI	LLE-SAFE	√			
19	2,7-Dimethyloctane	1072-16-8	2491		MS, RI	LLE-SAFE		√		
20	Dimethylmethane	74-98-6	2514		MS, RI	LLE-SAFE			√	√
21	2,6,10-Trimethyldodecane	3891-98-3	2709		MS, RI	LLE-SAFE		√		
22	Hexadecane	544-76-3	2805		MS, RI	LLE-SAFE	√	√	√	√
23	2-Methylpropane	75-28-5	2996		MS, RI	LLE-SAFE	√		√	
24	Butane	106-97-8	3044		MS, RI	LLE-SAFE	√	√		
25	1,3,6-Trioxocane	1779-19-7	3054		MS, RI	LLE-SAFE				√
26	Pentane	109-66-0	881		MS, RI	LLE-SAFE	√			
27	Bicyclo[2,1,0]pentane	185-94-4	889		MS, RI	LLE-SAFE	√	√	√	√
28	Ethylene oxide	75-21-8	893		MS, RI	LLE-SAFE	√		√	
29	Isobutylene oxide	558-30-5	910		MS, RI	LLE-SAFE	√	√	√	√
30	Cyclopropane	75-19-4	998		MS, RI	LLE-SAFE	√			√
31	1,3-Dimethoxybutane	10143-66-5		2290	MS, RI	SPME	√			
32	Acetaldehyde dipropyl acetal	105-82-8		2254	MS, RI	SPME		√	√	√
33	Methylcyclohexane	108-87-2			MS	LLE-SAFE	√	√	√	√
34	Tetratriacontane	14167-59-0			MS, aroma, S	LLE-SAFE	√	√		
35	Cycloheptane	291-64-5		1237	MS, RI	SPME	√			
36	Cyclooctane	292-64-8		1415	MS, RI	SPME	√			
37	1,2,3,4-Tetramethoxybutane	3011-85-6		2305	MS, RI	SPME	√	√	√	
38	1,1,3-Trimethylcyclohexane	3073-66-3			MS	LLE-SAFE			√	√
39	1,2-Dicyclohexylethane	3321-50-4			MS	LLE-SAFE		√	√	√
40	2,2-Dimethoxybutane	3453-99-4		2017	MS, RI	SPME			√	
41	Cholestane	481-21-0			MS, aroma, S	LLE-SAFE	√	√	√	√
42	11-Decyl-docosane	55401-55-3			MS	LLE-SAFE		√		
43	1-(1-ethoxyethoxy)-Butane	57006-87-8		671	MS, RI	SPME		√	√	
44	2,4-Dimethylhexane	589-43-5		1303	MS, RI	LLE-SAFE			√	
45	Heptacosane	593-49-7		2304	MS, RI	LLE-SAFE	√		√	√
46	1-Ethyl-2-propylcyclohexane	62238-33-9			MS	LLE-SAFE			√	
47	n-Pentadecane	629-62-9			MS	LLE-SAFE	√		√	√
48	n-Heneicosane	629-94-7			MS	LLE-SAFE			√	
49	n-Pentacosane	629-99-2			MS	LLE-SAFE		√		
50	Hexacosane	630-01-3			MS, aroma, S	LLE-SAFE	√		√	√
51	n-Octacosane	630-02-4			MS	LLE-SAFE	√	√	√	√
52	n-Nonacosane	630-03-5			MS	LLE-SAFE		√	√	√
53	Hentriacontane	630-04-6		2406	MS, RI	LLE-SAFE	√	√	√	√
54	n-Triacontane	638-68-6			MS	LLE-SAFE		√	√	√
55	Tetracosane	646-31-1			MS	LLE-SAFE	√	√		√
56	1,3,3-Trimethoxybutane	6607-66-5		2367	MS, RI	SPME	√		√	
57	Tetratetracontane	7098-22-8			MS, aroma, S	LLE-SAFE		√		√
Furans (42)										
1	2-Methyltetrahydrofuran-3-one	3188-00-9	1160		MS, RI	LLE-SAFE	√	√	√	√
2	2,2,5,5-Tetramethyltetrahydrofuran	15045-43-9	1307		MS, RI	LLE-SAFE			√	
3	Furfural	1998/1/1	1354	1303	MS, RI	LLE-SAFE, SPME	√	√	√	√
4	2-Acetyl furan	1192-62-7	1396		MS, RI, S	LLE-SAFE	√	√	√	√
5	5-Methyl-2-furaldehyde	620-02-0	1462		MS, RI, S	LLE-SAFE			√	
6	2-Propionylfuran	3194-15-8	1465		MS, RI	LLE-SAFE			√	
7	Furfuryl alcohol	98-00-0	1555	1375	MS, RI, aroma, S	LLE-SAFE, SPME	√	√	√	√
8	Tetrahydrofuran-2-ylmethylacetat	637-64-9	1566		MS, RI	LLE-SAFE		√		
9	2,2-Di(2-tetrahydrofuryl)propane	89686-69-1	1604		MS, RI	LLE-SAFE		√		
10	3-Methyl-2(5H)-furanone	22122-36-7	1605		MS, RI	LLE-SAFE	√	√	√	√
11	2(5H)-Furanone	497-23-4	1639		MS, RI	LLE-SAFE	√	√	√	√
12	2,5-Dimethyl-4-hydroxy-2H-furan-3-one	3658-77-3	1749	1865	MS, RI, aroma, S	LLE-SAFE	√	√	√	√
13	2-(1-Oxo-2-hydroxyethyl)furan	17678-19-2	1892		MS, RI	LLE-SAFE	√	√	√	√
14	5-Acetyltetrahydrofuran-2-one	29393-32-6	1945		MS, RI	LLE-SAFE	√	√	√	√
15	4-Hydroxy-5-methyl-3-furanone	19322-27-1	2009		MS, RI	LLE-SAFE		√	√	√
16	2H-Pyran-2,6(3H)-dione	5926-95-4	2009		MS, RI	LLE-SAFE			√	
17	2-Furaldehyde diethyl acetal	13529-27-6	2012		MS, RI	LLE-SAFE	√			
18	4-Methoxy-5H-furan-2-one	69556-70-3	2056		MS, RI	LLE-SAFE	√			
19	3-Furanmethanol	4412-91-3	2085		MS, RI	LLE-SAFE	√			
20	Ethyl 5-oxooxolane-2-carboxylate	1126-51-8	2116		MS, RI	LLE-SAFE	√	√	√	
21	Methyl 5-oxotetrahydrofuran-2-carboxylate	3885-29-8	2116		MS, RI	LLE-SAFE	√			
22	Tetrahydrofurfruyl alcohol	97-99-4	2155		MS, RI, S	LLE-SAFE			√	√
23	2-Furanmethanamine	617-89-0	2170		MS, RI	LLE-SAFE	√	√	√	√
24	2-Methyl-4H,5H-furo[3,2-c]pyridin-4-one	26956-44-5	2269		MS, RI	LLE-SAFE	√		√	√
25	5-Hydroxymethyl-2-furaldehyde	67-47-0	2387		MS, RI, aroma, S	LLE-SAFE	√	√	√	√
26	5-(Hydroxymethyl)dihydro-2(3H)-furanone	10374-51-3	2390		MS, RI	LLE-SAFE				√
27	5-(1-Piperidyl)furan-2-carbaldehyde	22868-60-6	2433		MS, RI	LLE-SAFE		√		
28	5-(Hydroxymethyl)furfuryl alcohol	1883-75-6	2469		MS, RI	LLE-SAFE	√	√	√	√
29	3-Furaldehyde	498-60-2	2514		MS, RI	LLE-SAFE	√		√	√
30	2,3-Dihydrofuran	1191-99-7		1116	MS, RI	SPME			√	
31	Furfuryl sulfide	13678-67-6			MS	LLE-SAFE		√		
32	4,6-Dimethoxy-2,3-dihydrobenzofuran-3-one	4225-35-8		2389	MS, RI	LLE-SAFE		√		
33	2,2′-Difurfuryl ether	4437-22-3		1190	MS, RI	SPME		√	√	√
34	2-Butylfuran	4466-24-4		1186	MS, RI, S	SPME			√	
35	Phthalan	496-14-0		1371	MS, RI	SPME			√	
36	2,3-Dihydrobenzofuran	496-16-2		2402	MS, RI	LLE-SAFE		√		
37	Methyl 2-furoate	611-13-2		2021	MS, RI, aroma, S	LLE-SAFE	√	√	√	√
38	2,5-Furandicarboxaldehyde	823-82-5		1999	MS, RI	LLE-SAFE	√	√	√	√
39	2-Furancarboxylic acid	88-14-2		2249	MS, RI, aroma, S	LLE-SAFE	√	√		√
40	3-Methylfuran	930-27-8		1206	MS, RI	SPME	√	√		
41	5-Methoxy-2,3-dihydrobenzofuran-3-acetic acid	93198-71-1			MS	LLE-SAFE			√	
42	2-Methyltetrahydrofuran	96-47-9		690	MS, RI	SPME		√		
Aromatic compounds (41)										
1	Styrene	100-42-5	1152		MS, RI	LLE-SAFE		√	√	√
2	Benzocyclobutene	694-87-1	1152		MS, RI	LLE-SAFE		√		
3	Benzaldehyde	100-52-7	1411		MS, RI, S	LLE-SAFE			√	
4	Benzeneacetaldehyde	122-78-1	1531		MS, RI, S	LLE-SAFE	√	√	√	√
5	Ethyl benzoate	93-89-0	1558		MS, RI, S	LLE-SAFE	√			
6	2-Phenylethyl acetate	103-45-7	1706	1444	MS, RI, aroma, S	LLE-SAFE, SPME	√	√	√	√
7	Benzyl alcohol	100-51-6	1767		MS, RI, S	LLE-SAFE	√	√	√	√
8	Phenethyl alcohol	1960/12/8	1801	1471	MS, RI, aroma, S	LLE-SAFE, SPME	√	√	√	√
9	Toluene	108-88-3	1803		MS, RI	LLE-SAFE	√	√	√	√
10	2,6-Di-tert-butyl-4-methylphenol	128-37-0	1807		MS, RI, S	LLE-SAFE	√	√	√	√
11	DL-β-Ethylphenethyl alcohol	2035-94-1	1876		MS, RI	LLE-SAFE	√			
12	Phenol	108-95-2	1894	2009	MS, RI, S	LLE-SAFE	√	√	√	√
13	3-Phenoxypropionic acid	7170-38-9	1964		MS, RI	LLE-SAFE	√			
14	2-Methoxy-4-vinylphenol	7786-61-0	2083	1647	MS, RI, aroma, S	LLE-SAFE, SPME	√	√	√	√
15	3-Hydroxy-4-phenylbutane-2-one	5355-63-5	2146		MS, RI	LLE-SAFE	√	√	√	√
16	Ethyl 2-hydroxy-3-phenylpropanoate	15399-05-0	2164		MS, RI	LLE-SAFE	√	√	√	√
17	1,3-Dioxolane,2-(1-phenylethyl)	4362-22-5	2165		MS, RI	LLE-SAFE	√		√	
18	2,4-Di-tert-butylphenol	96-76-4	2207	2309	MS, RI, aroma, S	LLE-SAFE	√	√	√	√
19	1-Phenyl-2-propanone	103-79-7	2219		MS, RI	LLE-SAFE			√	√
20	4-Vinylphenol	2628-17-3	2281		MS, RI	LLE-SAFE	√	√	√	√
21	1,3-Diphenylpropan-2-ol	5381-92-0	2400		MS, RI	LLE-SAFE	√	√	√	√
22	3-Hydroxy-3-phenylbutan-2-one	3155/1/9	2410		MS, RI	LLE-SAFE		√	√	√
23	α-Methylstyrene	98-83-9	2679		MS, RI	LLE-SAFE	√	√	√	√
24	4-n-Propylphenol	645-56-7	2703		MS, RI, S	LLE-SAFE				√
25	4-(2-Acetoxy-ethyl)phenol	58556-55-1	2821		MS, RI	LLE-SAFE	√	√	√	√
26	p-Hydroxybenzaldehyde	123-08-0	2836		MS, RI, S	LLE-SAFE			√	
27	4-Methoxyresorcinol	6100-60-3	2846		MS, RI	LLE-SAFE		√	√	√
28	2-Methoxyhydroquinone	824-46-4	2846		MS, RI, S	LLE-SAFE		√	√	√
29	4-Hydroxyphenylethanol	501-94-0	2893		MS, RI, aroma, S	LLE-SAFE	√	√	√	√
30	Benzeneacetic acid	103-82-2			MS, aroma, S	LLE-SAFE	√	√	√	√
31	Resorcinol	108-46-3		2084	MS, RI, aroma, S	LLE-SAFE				√
32	1-(2-Hydroxy-5-methylphenyl)ethanone	1450-72-2		1647	MS, RI	SPME		√		√
33	(R)-(+)-1-Phenylethanol	1517-69-7		1220	MS, RI	SPME	√	√		√
34	4-Phenylbutanal	18328-11-5		1428	MS, RI	SPME		√		√
35	4-Methoxyphenoxyacetic acid	1877-75-4			MS	LLE-SAFE	√		√	√
36	4-Hydroxyphenoxyacetic acid	1878-84-8			MS	LLE-SAFE			√	
37	3,5-Dimethoxyphenol	500-99-2			MS, S	LLE-SAFE		√		
38	2,3,5,6-Tetramethyl-pheno	527-35-5		1645	MS, RI, S	SPME	√		√	
39	2,5-Di-tert-butylphenol	5875-45-6		1701	MS, RI	SPME	√	√	√	√
40	Benzoic acid	65-85-0		2449	MS, RI, aroma, S	LLE-SAFE	√	√	√	√
41	2,5-Dimethylphenol	95-87-4		2196	MS, RI, aroma, S	LLE-SAFE			√	
Ketones (37)										
1	3-Penten-2-one	3102-33-8	1026		MS, RI	LLE-SAFE		√		
2	3-Penten-2-one	625-33-2	1026		MS, RI	LLE-SAFE		√	√	
3	Cyclopentanone	120-92-3	1082		MS, RI	LLE-SAFE	√			√
4	2,2,5-Trimethylhexane-3,4-dione	20633-03-8	1105		MS, RI	LLE-SAFE				√
5	3-Hydroxy-2-butanone	513-86-0	1179	1277	MS, RI, S	LLE-SAFE	√	√	√	√
6	Hydroxyacetone	116-09-6	1191		MS, RI	LLE-SAFE	√	√	√	√
7	2-Cyclopenten-1-one	930-30-3	1248		MS, RI	LLE-SAFE	√	√	√	√
8	2-Hydroxy-3-pentanone	5704-20-1	1250		MS, RI	LLE-SAFE		√	√	
9	4-Hydroxy-4-methyl-2-pentanone	123-42-2	1254		MS, RI	LLE-SAFE	√	√	√	√
10	4-Hydroxy-3-hexanone	4984-85-4	1302		MS, RI	LLE-SAFE			√	
11	4-Hydroxy-2-pentanone	4161-60-8	1350		MS, RI	LLE-SAFE		√	√	√
12	2-Butanone	78-93-3	1359		MS, RI	LLE-SAFE		√	√	
13	2,5-Hexanedione	110-13-4	1394		MS, RI	LLE-SAFE		√		√
14	4-Hydroxy-2-butanone	590-90-9	1427	1737	MS, RI, aroma, S	LLE-SAFE	√	√	√	√
15	3-Hydroxy-3-methyl-2-butanone	115-22-0	1451		MS, RI	LLE-SAFE			√	√
16	Isophorone	78-59-1	1483		MS, RI	LLE-SAFE	√	√	√	√
17	3-Methylcyclopentane-1,2-dione	765-70-8	1726		MS, RI	LLE-SAFE	√		√	√
18	3,4-Dihydroxy-2-butanone	57011-15-1	1819		MS, RI	LLE-SAFE	√		√	√
19	1,3-Dioxolan-2-one	96-49-1	1827		MS, RI	LLE-SAFE	√		√	
20	1-Hydroxy-2-pentanone	64502-89-2	1978		MS, RI	LLE-SAFE			√	
21	4-Methyl-2,3-pentanedione	7493-58-5	2003		MS, RI	LLE-SAFE	√			
22	2-Ethyl-cyclopentanone	4971-18-0	2009		MS, RI	LLE-SAFE	√			
23	2,3-Butanedione	431-03-8	2038		MS, RI, S	LLE-SAFE			√	
24	4-Acetoxy-2-butanone	10150-87-5	2424		MS, RI	LLE-SAFE	√	√	√	√
25	4-Hydroxy-β-damascone	102488-09-5	2425		MS, RI	LLE-SAFE		√	√	√
26	4-(Hydroxymethyl)-1,3-dioxolan-2-one	931-40-8	2716		MS, RI	LLE-SAFE	√		√	
27	Acetone	67-64-1	917		MS, RI	LLE-SAFE	√	√	√	√
28	2,3-Pentanedione	600-14-6	982		MS, RI	LLE-SAFE			√	√
29	2,2,6-Trimethylcyclohexanone	2408-37-9			MS	LLE-SAFE	√			√
30	2-Ethyl-3-methoxy-2-cyclopentenone	25112-86-1			MS	LLE-SAFE				√
31	3-Methyl-4-nonanone	35778-39-3		681	MS, RI	SPME	√		√	
32	3,4,5,6-Tetrahydropseudoionone	4433-36-7			MS	LLE-SAFE			√	
33	5-Methyl-3-heptanone	541-85-5		2215	MS, RI	SPME		√		
34	4-Octanone	589-63-9		1150	MS, RI	SPME	√	√	√	√
35	3-Methyl-cyclohexanone	591-24-2		1188	MS, RI	SPME	√			
36	(Z)-β-Ionone	79-77-6			MS, aroma, S	LLE-SAFE		√		√
37	Verbenone	80-57-9		1645	MS, RI	SPME			√	
Acids (33)										
1	2-Methylvaleric acid	594-61-6	1411		MS, RI, S	LLE-SAFE	√	√	√	√
2	Acetic acid	64-19-7	1473	1296	MS, RI, aroma, S	LLE-SAFE, SPME	√	√	√	√
3	4-Methyl-3-pentenoic acid	504-85-8	1478		MS, RI	LLE-SAFE	√	√	√	√
4	3-Hydroxypropionic acid	503-66-2	1489		MS, RI	LLE-SAFE			√	
5	Malonic acid	141-82-2	1715		MS, RI	LLE-SAFE			√	
6	Butanoic acid	107-92-6	1718		MS, RI	LLE-SAFE		√		
7	Hexanoic acid	142-62-1	1824	1441	MS, RI, aroma, S	LLE-SAFE, SPME	√	√	√	√
8	4-Methylvaleric acid	646-07-1	1832		MS, RI	LLE-SAFE				√
9	Pentanoic acid	109-52-4	1870		MS, RI, S	LLE-SAFE	√	√	√	√
10	4-Methylpent-4-enoic acid	1001-75-8	2006		MS, RI	LLE-SAFE			√	
11	Octanoic acid	124-07-2	2006	1544	MS, RI, aroma, S	LLE-SAFE, SPME	√	√	√	√
12	2-Methyl-2-pentenoic acid	3142-72-1	2011	1199	MS, RI, S	LLE-SAFE, SPME	√	√	√	√
13	3-Methylbutanoic acid	503-74-2	2062	1379	MS, RI, aroma, S	LLE-SAFE, SPME	√	√	√	√
14	2-Propynyl acetate	627-09-8	2112		MS, RI	LLE-SAFE	√	√		√
15	2-Oxopropionic acid	127-17-3	2229		MS, RI	LLE-SAFE	√	√	√	√
16	Decanoic acid	334-48-5	2322	1684	MS, RI, aroma, S	LLE-SAFE, SPME	√	√	√	√
17	Nonanoic acid	112-05-0	2342		MS, RI, S	LLE-SAFE				√
18	L-Lactic acid	79-33-4	2947		MS, RI	LLE-SAFE		√		
19	4-Methyl-2-pentenoic acid	10321-71-8		1955	MS, RI	LLE-SAFE			√	√
20	DL-3-Methylvaleric acid	105-43-1		1373	MS, RI, S	SPME		√		
21	Heptanoic acid	111-14-8		1619	MS, RI, S	SPME	√		√	√
22	Hendecanoic acid	112-37-8		1492	MS, RI	SPME	√		√	√
23	Elaidic acid	112-79-8			MS, aroma, S	LLE-SAFE	√	√		√
24	3-Ethylheptanoic acid	14272-47-0		1629	MS, RI	SPME	√	√	√	
25	Dodecanoic acid	143-07-7		2486	MS, RI, aroma, S	LLE-SAFE	√	√	√	√
26	2-(2-Methoxyethoxy)acetic acid	16024-56-9		2311	MS, RI	SPME	√			
27	4-Methoxybutyric acid	29006-02-8		2321	MS, RI	SPME	√		√	√
28	Crotonic acid	3724-65-0		2395	MS, RI	LLE-SAFE		√		
29	trans-3-Hexenoic acid	4219-24-3		2128	MS, RI, aroma, S	LLE-SAFE	√	√	√	√
30	3-Ethoxypropionic acid	4324-38-3		1859	MS, RI	SPME	√			√
31	Palmitic acid	1957/10/3			MS, aroma, S	LLE-SAFE	√	√	√	√
32	Tridecanoic acid	638-53-9		1620	MS, RI, S	SPME	√	√	√	√
33	Citric acid	77-92-9		1730	MS, RI	SPME				√
Sulfur-containing compounds (27)										
1	Thiazole	288-47-1	1145		MS, RI	LLE-SAFE	√			
2	Acetyl Sulfide	3232-39-1	1164		MS, RI	LLE-SAFE		√	√	√
3	2-(Methylthio)ethanol	5271-38-5	1422		MS, RI, S	LLE-SAFE	√	√	√	√
4	Dimethyl sulfoxide	67-68-5	1467		MS, RI, S	LLE-SAFE	√	√	√	√
5	3-(Methylthio)-1-propanol	505-10-2	1610	1714	MS, RI, aroma, S	LLE-SAFE, SPME	√	√	√	√
6	Thioacetic acid	507-09-5	1630		MS, RI, S	LLE-SAFE	√			√
7	S-Ethyl ethanethioate	625-60-5	1704		MS, RI, S	LLE-SAFE	√			
8	Dimethyl sulfone	67-71-0	1785		MS, RI, S	LLE-SAFE	√	√	√	√
9	4-methyl-5-hydroxyethyl thiazole	137-00-8	2197		MS, RI	LLE-SAFE	√	√		√
10	Methyl p-tolyl sulfone	3185-99-7	2504		MS, RI	LLE-SAFE				√
11	Methyl mercaptan	74-93-1	893		MS, RI, S	LLE-SAFE	√	√	√	√
12	Tetrahydrothiophene	110-01-0		1679	MS, RI	SPME	√	√		
13	Thiophene	110-02-1		1031	MS, RI, aroma, S	LLE-SAFE, SPME	√	√	√	√
14	2,3-Dihydrothiophene	1120-59-8		2395	MS, RI	LLE-SAFE		√		
15	2-Thiopheneacetic acid	1918-77-0		1187	MS, RI	SPME		√	√	√
16	(Butylthio)acetic acid	20600-61-7		1989	MS, RI	SPME		√		
17	[(2-Methylpropyl)thio]acetic acid	20600-62-8		2282	MS, RI	SPME	√			
18	3-(n-Butylsulphanyl)propionic acid	22002-73-9		2383	MS, RI	SPME		√		
19	2-Pyrrolidinethione	2295-35-4		1219	MS, RI	SPME			√	√
20	Isothiazole	288-16-4		652	MS, RI	LLE-SAFE			√	√
21	2-Acetyl-2-thiazoline	29926-41-8		1631	MS, RI	SPME		√		
22	4-Methyl-5-acetyl thiazole	38205-55-9		1349	MS, RI	SPME				√
23	Thiazolidine-4-carboxylic acid	444-27-9		2219	MS, RI	SPME	√	√	√	
24	Thiazolidine	504-78-9		1893	MS, RI	SPME				√
25	Diethyl sulfite	623-81-4		2166	MS, RI	SPME	√	√		√
26	5-Methylthio-1,3,4-thiadiazole-2-thiol	6264-40-0		2158	MS, RI	SPME				√
27	Divinyl sulfide	627-51-0		2399	MS, RI	LLE-SAFE			√	
Lactones (24)										
1	α-Methyl-γ-butyrolactone	1679-47-6	1476		MS, RI	LLE-SAFE	√	√	√	√
2	4,4-Dimethylbut-2-en-4-olide	20019-64-1	1496		MS, RI	LLE-SAFE	√	√	√	√
3	γ-Valerolactone	108-29-2	1496		MS, RI, S	LLE-SAFE	√	√	√	√
4	3-Methyl-4-butanolide	1679-49-8	1500		MS, RI	LLE-SAFE	√	√	√	√
5	γ-Butyrolactone	96-48-0	1512		MS, RI, S	LLE-SAFE	√	√	√	√
6	γ-Hexanolactone	695-06-7	1589		MS, RI, S	LLE-SAFE	√	√	√	√
7	δ-Hexanolide	823-22-3	1679		MS, RI, S	LLE-SAFE	√	√	√	√
8	δ-Valerolactone	542-28-9	1688		MS, RI, S	LLE-SAFE	√	√	√	√
9	3-Methyl-2-buten-4-olide	6124-79-4	1769	1903	MS, RI	LLE-SAFE	√	√	√	√
10	γ-Octalactone	104-50-7	1803		MS, RI, S	LLE-SAFE	√	√	√	√
11	3-Methyl-2-penten-5-olide	2381-87-5	1903		MS, RI	LLE-SAFE	√	√	√	√
12	4-Nonanolide	104-61-0	1915		MS, RI, S	LLE-SAFE	√	√	√	√
13	L-(+)-Pantolactone	5405-40-3	1917		MS, RI	LLE-SAFE	√	√		√
14	D-(−)-Pantolactone	599-04-2	1917		MS, RI	LLE-SAFE	√	√	√	√
15	DL-Pantoyl Lacyone	79-50-5	1917		MS, RI	LLE-SAFE		√		
16	2-Hydroxy-γ-butyrolactone	19444-84-9	2053		MS, RI	LLE-SAFE	√	√	√	√
17	Dihydroactinidiolide	15356-74-8	2224		MS, RI, S	LLE-SAFE	√	√	√	√
18	(R)-Dihydro-actinidiolide	17092-92-1	2224		MS, RI	LLE-SAFE				√
19	5-Hydroxy-4-pentanolide	32780-06-6	2364		MS, RI	LLE-SAFE	√	√	√	√
20	DL-Mevalonolactone	674-26-0	2428		MS, RI	LLE-SAFE	√	√	√	√
21	(±)-3-Hydroxy-4-butanolide	5469-16-9	2479		MS, RI, aroma, S	LLE-SAFE	√	√	√	√
22	β-Butyrolactone	3068-88-0	2531		MS, RI	LLE-SAFE	√			
23	Costunolide	553-21-9			MS, aroma, S	LLE-SAFE		√	√	
24	γ-Decanolactone	706-14-9		2040	MS, RI, aroma, S	LLE-SAFE	√	√	√	√
Aldehydes (19)										
1	3-Methyl-2-butenal	107-86-8	1096		MS, RI	LLE-SAFE	√	√	√	
2	Nonanal	124-19-6	1289		MS, RI, S	LLE-SAFE		√	√	
3	1,3,5,7-Tetroxane	293-30-1	1418		MS, RI	LLE-SAFE	√	√	√	√
4	Methyl glyoxal	78-98-8	1458		MS, RI	LLE-SAFE	√	√	√	√
5	Glutaraldehyde	111-30-8	1545	2353	MS, RI	LLE-SAFE, SPME		√	√	√
6	(Z)-2-Methyl-2-butenal	1115-11-3	1636		MS, RI	LLE-SAFE				√
7	Methacrolein	78-85-3	1686		MS, RI	LLE-SAFE	√	√	√	√
8	2-Ethyl-4-pentenal	5204-80-8	2038		MS, RI	LLE-SAFE	√			
9	Methoxy acetaldehyde	10312-83-1	2216		MS, RI	LLE-SAFE	√		√	√
10	cis-6-Nonenal	2277-19-2	2517		MS, RI	LLE-SAFE				√
11	Glyoxylic acid	298-12-4	2643		MS, RI	LLE-SAFE		√		
12	2,2-Dimethylocta-3,4-dienal	590-71-6	2796		MS, RI	LLE-SAFE				√
13	3-Hydroxybutyraldehyde	107-89-1	2929		MS, RI	LLE-SAFE	√	√	√	√
14	Acetaldehyde	75-07-0	890		MS, RI, S	LLE-SAFE	√	√	√	√
15	Hexanal	66-25-1	993		MS, RI	LLE-SAFE		√	√	
16	trans-2-Nonenal	18829-56-6		1414	MS, RI, S	SPME	√	√		
17	Isovaleraldehyde	590-86-3		2047	MS, RI, S	SPME	√			
18	(E,E)-2,4-Nonadienal	5910-87-2		1123	MS, RI	SPME	√			√
19	Hexadecanal	629-80-1			MS	LLE-SAFE	√		√	
Acetals (2)										
1	2-Benzyl-1,3-dioxolane	101-49-5	2165		MS, RI	LLE-SAFE	√		√	
2	Tetraethylene glycol	112-60-7	2597	2144	MS, RI	LLE-SAFE, SPME	√	√	√	√
Others (37)										
1	Myrcene	123-35-3	1066	1056	MS, RI, S	LLE-SAFE, SPME	√		√	√
2	Dimethyl ether	115-10-6	1110		MS, RI	LLE-SAFE			√	√
3	Acetic anhydride	108-24-7	1134		MS, RI	LLE-SAFE	√	√		√
4	1,3,5,7-Cyclooctatetraene	629-20-9	1152		MS, RI	LLE-SAFE				√
5	3,5-Dimethylhex-1-ene	7423-69-0	1659		MS, RI	LLE-SAFE			√	
6	Ethoxyacetylene	927-80-0	1676		MS, RI	LLE-SAFE		√		
7	1,5-Heptadien-3-yne	3511-27-1	1806		MS, RI	LLE-SAFE	√	√	√	
8	Isobutyl anhydride	97-72-3	2012		MS, RI	LLE-SAFE		√	√	
9	2-Methyl-2-butene	513-35-9	2039		MS, RI, S	LLE-SAFE	√	√	√	√
10	3-Methylene-nonane	51655-64-2	2039		MS, RI	LLE-SAFE	√			
11	Valeric anhydride	2082-59-9	2116		MS, RI	LLE-SAFE				√
12	2-Methylsuccinic anhydride	4100-80-5	2264		MS, RI	LLE-SAFE	√	√	√	√
13	1-Docosene	1599-67-3	2481		MS, RI	LLE-SAFE				√
14	1-Hexadecene	629-73-2	2481		MS, RI	LLE-SAFE				√
15	1,4-Dioxane	123-91-1	2713		MS, RI	LLE-SAFE		√		
16	Cyclopentene	142-29-0	889		MS, RI	LLE-SAFE				√
17	Propene	115-07-1	908		MS, RI	LLE-SAFE	√	√	√	√
18	1,3-Butadiyne	460-12-8	918		MS, RI	LLE-SAFE				√
19	1-Buten-3-yne	689-97-4	921		MS, RI	LLE-SAFE		√		
20	1-Nonyne	3452/9/3		1115	MS, RI	SPME				√
21	cis-2-Octene	7642/4/8		1292	MS, RI	SPME	√	√		√
22	Diisopropyl ether	108-20-3		2325	MS, RI	SPME	√		√	√
23	2-Pentene	109-68-2		606	MS, RI, aroma, S	LLE-SAFE		√	√	
24	3-Ethyl-3-hexene	16789-51-8		1282	MS, RI	SPME				√
25	β-Pinene	18172-67-3		1040	MS, RI, S	SPME	√	√	√	
26	2-Methyl-2-hexene	2738-19-4		1412	MS, RI	SPME				√
27	(E)-2-Hexene	4050-45-7		1175	MS, RI	SPME			√	
28	Dodecyl ether	4542-57-8			MS	LLE-SAFE	√		√	√
29	3-Methyl-1-butene	563-45-1		605	MS, RI	LLE-SAFE		√	√	√
30	1,2-Butadiene	590-19-2		613	MS, RI	LLE-SAFE				√
31	1-Heptene	592-76-7		1228	MS, RI, S	SPME	√	√	√	√
32	1-Octadecyne	629-89-0			MS	LLE-SAFE	√		√	√
33	1,1-Diethoxy-3,7-dimethylocta-2,6-diene	7492-66-2		1216	MS, RI	SPME	√	√		
34	2-Methyl-1-pentene	763-29-1		1050	MS, RI	SPME	√	√		√
35	2-Methyl-1,3-butadiene	78-79-5		605	MS, RI	LLE-SAFE	√			
36	1-Undecene	821-95-4		1349	MS, RI, S	SPME	√	√	√	√
37	Vinyl isopropyl ether	926-65-8		1044	MS, RI	SPME		√	√	

Note: √ indicates the presence of the compound in the corresponding brand.

**Table A4:** GC-O-MS sniffing results of volatile compounds in different brands of lager beer.

No.	Compound Name	CAS	Sniffing Description	YJ	BW	HR	QD
1	4-Vinylguaiacol	7786-61-0	Coffee bean, nut shell, sour	4	3	1.5	3
2	γ-Decalactone	706-14-9	Sweet, floral, gardenia, creamy	4	4.5	4	4
3	Phenylacetic acid	103-82-2	Floral, rose, sweet, grassy	3.5	4	2.5	2
4	Isovaleric acid	503-74-2	Sour, durian-like	3.5	3	3.5	2.5
5	2-Phenylethyl acetate	103-45-7	Sweet, floral, fruity	3	2.5	3.5	1
6	Acetic acid	64-19-7	Sour, muddy	3	2.5	4	3
7	Octanoic acid	124-07-2	Grassy, vegetal	3	2.5	2.5	2
8	Hexyl butyrate	2639-63-6	Sweet, floral, honey-like	2.5	2	2	1.5
9	6-Methyl-5-hepten-2-one	3658-77-3	Caramel, sweet	2.5	3	2.5	2
10	Phenylethanol	1960/12/8	Rose-like, sweet	2.5	3.5	3.5	2.5
11	Pineapple alcohol	505-10-2	Sour, muddy	2.5	2.5	3	3
12	2,3-Butanediol	513-85-9	Roasted, dark wheat	2	1	-	-
13	Hexanoic acid	142-62-1	Lemon-like, sour, bitter	2	2	2.5	0.5
14	Decanoic acid	334-48-5	Sour, hoppy, grassy	2	1.5	1.5	0.5
15	2-Acetylpyrrole	1072-83-9	Malty, sweet, caramel	1.5	2.5	1.5	1
16	Furoic acid	88-14-2	Rooty, wheaty	1.5	2	-	1.5
17	4-Hydroxyphenylethanol	501-94-0	Floral, sweet, fruity	1.5	2	3	1
18	Ethyl propionate	105-37-3	Floral, fruity, strawberry-like	1	2	1.5	1
19	Hexacosane	630-01-3	Floral, honey-like	1	2	0.5	-
20	Resorcinol	108-46-3	Burnt, sweet	-	2	-	-
21	trans-Oleic acid	112-79-8	Dark wheat	-	2	-	2
22	5-Methylpyrrole-2-carboxaldehyde	1192-79-6	Rooty	1.5	1.5	-	-
23	trans-3-Hexenoic acid	4219-24-3	Floral, sweet, burnt	1.5	1.5	1	-
24	Isoamyl alcohol	123-51-3	Nut shell, roasted	1	1.5	2	1
25	Furfuryl alcohol	98-00-0	Sour, putrid	1	1.5	0.5	-
26	Palmitic acid	1957/10/3	Dark wheat	1	1.5	-	-
27	(±)-3-Hydroxy-4-butyrolactone	5469-16-9	Floral, woody	1	1.5	1	1.5
28	Monoethyl succinate	1070-34-4	Malty	-	1.5	-	-
29	2,4-Di-tert-butylphenol	96-76-4	Roasted, nut shell, yeasty	1	1	1	1.5
30	Ethyl hexanoate	123-66-0	Sweet, fruity, floral	0.5	1	1.5	1.5
31	4-Hydroxy-2-butanone	590-90-9	Floral, oily, sweet	0.5	1	2	0.5
32	Methyl octanoate	111-12-6	Dusty, moldy, bitter	-	1	1	-
33	6-(Ethoxycarbonyl)nicotinic acid	17874-78-1	Smoky	-	1	-	-
34	(Z)-β-Ionone	79-77-6	Floral, sweet	-	1	-	0.5
35	Cholestane	481-21-0	Roasted, dark wheat	1	0.5	-	-
36	1,2-Propanediol	57-55-6	Nut shell	-	0.5	-	-
37	Isoamyl acetate	123-92-2	Floral	-	0.5	-	-
38	Tetratetracontane	7098-22-8	Nut shell	-	0.5	-	-
39	Methyl decanoate	110-42-9	Apple-like, sweet, floral	1.5	-	-	1
40	(Z)-9-Octadecenamide	301-02-0	Sweet, yeasty	1	-	-	1
41	2,6-Di-tert-butylpyridine	585-48-8	Ashy	1	-	-	-
42	5-Hydroxymethylfurfural	67-47-0	Yeasty	1	-	-	-
43	Benzoic acid	65-85-0	Ashy	1	-	-	-
44	Lauric acid	143-07-7	Fruity	1	-	-	-
45	2-Hexyl-1-decanol	2425-77-6	Floral	0.5	-	0.5	-
46	Thiophene	110-02-1	Garlic, oily	-	-	2.5	-
47	2-Hydroxypyridine	142-08-5	Hoppy, bitter	-	-	2	-
48	2-Pentene	109-68-2	Burnt, plastic-like	-	-	2	-
49	2,5-Dimethylphenol	95-87-4	Nut shell	-	-	1	-
50	Methyl furoate	611-13-2	Nut shell	-	-	0.5	-
51	N-Butylacetamide	1119-49-9	Sweet	-	-	-	1
52	Tetratriacontane	14167-59-0	Sweet	-	-	-	2
53	Costunolide	553-21-9	Floral	-	-	-	0.5

**Table A5:** Odor and taste activity values (OAV and TAV) of flavor compounds in different lager beer samples.

No.	Compound Name	Odor Threshold (μg/L)	OAV				Taste Threshold (μg/L)	TAV			
			YJ	QD	HR	BW		YJ	QD	HR	BW
1	Isoamyl acetate	0.13	2,046,526	1,217,009	4,338,735	2,821,490	2.63	102,326	60,850	216,937	141,074
2	Ethyl hexanoate	4.35	8113	6941	4690	10,366	4.35	8113	6941	4690	10,366
3	Ethyl propanoate	8.88	4468	2403	6090	5094	8.88	4468	2403	6090	5094
4	Hexyl butanoate	172.75	1354	718	1797	1547	—	—	—	—	—
5	Methyl decanoate	3.75	19,448	16,822	29,701	45,970	871.00	84	72	128	198
6	Ethyl octanoate	16.73	105	30	79	97	0.09	20,170	5840	15,231	18,693
7	Ethyl decanoate	4.31	249	—	320	634	17.24	62	—	80	158
8	Isoamyl butyrate	12.93	3658	—	—	—	—	—	—	—	—
9	Ethyl trans-2-decenoate	10,000.00	—	—	<1	—	—	—	—	—	—
10	Octyl acetate	0.04	99	22	—	—	182.28	22	5	—	—
11	Ethyl stearate	0.53	26	23	12	27	—	—	—	—	—
12	Ethyl isovalerate	0.10	—	31,502	—	—	0.17	—	17,326	—	—
13	Ethyl myristate	3440.00	1	<1	<1	1	—	—	—	—	—
14	Ethyl lactate	51,550.00	<1	<1	<1	<1	257,750.00	<1	<1	<1	<1
15	Ethyl acetate	50.00	11,099	8987	10,306	7351	6765.00	82	66	76	54
16	Ethyl heptanoate	1.65	350	409	254	623	147.90	4	5	3	7
17	Hexyl acetate	20.00	286	392	574	551	34.80	164	225	330	317
18	Diethyl succinate	104,700.00	<1	<1	<1	<1	1,094,115.00	<1	<1	<1	<1
19	Butyl acetate	51.04	—	—	13	14	88.00	—	—	8	8
20	Ethyl 3-hydrobutyrate	1000.00	—	—	<1	<1	—	—	—	—	—
21	Ethyl nonanoate	326.48	7	2	9	16	1039.20	2	1	3	5
22	Ethyl hydrogen succinate	1,141,000.00	<1	<1	<1	<1	1,369,200.00	<1	<1	<1	<1
23	Ethyl valerate	5.08	774	679	596	623	787.50	5	4	4	4
24	Methyl 2-octynate	23.00	—	—	721	575	—	—	—	—	—
25	(−)-Ethyl L-lactate	—	—	—	—	—	—	—	—	—	—
26	2-Hydroxyethyl acetate	—	—	—	—	—	—	—	—	—	—
27	Propyl acetate	1776.00	<1	<1	—	—	710.40	1	<1	—	—
28	2,3-Dihydroxypropyl acetate	—	—	—	—	—	1,616,040.00	<1	<1	<1	<1
29	Methyl propionate	4209.00	—	<1	—	—	53.07	—	73	—	—
30	Isopropyl formate	—	—	—	—	—	—	—	—	—	—
31	Ethyl dodecanoate	5091.70	1	—	<1	—	284.79	24	—	17	—
32	Ethyl 3-hexenoate	10,000.00	3	—	<1	<1	—	—	—	—	—
33	Ethyl butyrylacetate	—	—	—	—	—	—	—	—	—	—
34	Ethyl 3-methylvalerate	—	—	—	—	—	—	—	—	—	—
35	Ethyl undecanoate	—	—	—	—	—	859.00	56	—	3	—
36	(+)-Diethyl L-tartrate	—	—	—	—	—	—	—	—	—	—
37	2,3-Butaneiol	100,200.00	2	<1	4	5	50,100.00	5	1	8	11
38	3-Methyl-1-butanol	792.82	710	531	1045	913	202.25	2785	2082	4098	3579
39	1,2,3-Propanetriol	25,000,000.00	<1	<1	<1	<1	6,562,500.00	<1	<1	<1	<1
40	2-Methyl-1-propanol	11.00	14,796	12,246	15,732	6183	8.83	18,426	15,251	19,592	7700
41	Diisobutylcarbinol	1051.70	—	—	3	2	—	—	—	—	—
42	1-Butanol	3483.00	3	<1	<1	<1	162,000.00	<1	<1	<1	<1
43	1-Octanol	104.04	4	5	11	8	44.66	10	11	27	18
44	1-Pentanol	121.81	2030	1596	2460	2104	64,880.00	4	3	5	4
45	3-Methyl-3-buten-1-ol	466.70	<1	<1	<1	<1	—	—	—	—	—
46	α-Terpineol	1116.00	<1	<1	<1	—	279.00	2	2	1	—
47	2-Ethyl-1-hexanol	21,227.00	—	—	—	<1	—	—	—	—	—
48	4-Methyl-1-pentanol	673.22	1	—	—	99	—	—	—	—	—
49	1,2-Propylene glycol	352,240.00	<1	<1	<1	<1	1,450,400.00	<1	<1	<1	<1
50	2-Hexyl-1-decanol	—	—	—	—	—	—	—	—	—	—
51	1-Hexanol	318.27	10	24	13	10	162.80	20	47	26	20
52	Linalool	941.34	71	6	3	—	23.49	2854	242	114	—
53	1-Decanol	642.48	—	16	16	—	19.07	—	548	536	—
54	Diethylene glycol monoethyl ether	—	—	—	—	—	47,952.00	<1	<1	<1	<1
55	(−)-Isolongifolol	—	—	—	—	—	—	—	—	—	—
56	Phytol	—	—	—	—	—	—	—	—	—	—
57	β-Eudesmol	—	—	—	—	—	—	—	—	—	—
58	Guaiol	—	—	—	—	—	—	—	—	—	—
59	2,6-Dimethyl-5,7-octadien-2-ol	—	—	—	—	—	—	—	—	—	—
60	Phenethyl alcohol	397.80	5968	3462	8289	4426	50,100.00	5	1	8	11
61	2-Phenylethyl acetate	257.58	100	16	231	72	102,000.00	23	14	32	17
62	Phenol	59,000.00	<1	<1	<1	<1	20.64	1248	205	2877	894
63	Benzeneacetaldehyde	6.80	4206	5867	990	633	5890.50	<1	<1	1	1
64	Benzyl alcohol	2660.80	<1	<1	<1	<1	9.71	2944	4107	693	443
65	Ethyl benzoate	58.06	615	—	—	—	5747.50	<1	<1	<1	<1
66	Benzaldehyde	783.93	—	—	2	—	313,500.00	<1	—	—	—
67	2,4-Di-tert-butylphenol	443.50	18	157	73	88	313.20	—	—	5	—
68	2-Methoxy-4-vinylphenol	20.69	1128	1171	1740	1290	—	—	—	—	—
69	2,6-Di-tert-butyl-4-methylphenol	1048.00	500	274	572	327	21.78	1072	1113	1653	1226
70	Benzoic acid	1080.00	102	126	133	82	—	—	—	—	—
71	p-Hydroxyphenylethanol	—	—	—	—	—	313,200.00	<1	<1	<1	<1
72	Benzeneacetic acid	12,972.00	8	6	6	7	—	—	—	—	—
73	Resorcinol	7620.00	—	—	—	<1	108.10	936	696	684	846
74	2,5-Dimethylphenol	388.40	—	—	6	—	93,980.00	—	—	—	<1
75	α-Methylstyrene	—	—	—	—	—	485.50	—	—	5	—
76	1-(2-Hydroxy-5-methylphenyl)ethanone	—	—	—	—	—	—	—	—	—	—
77	(R)-(+)-1-Phenylethanol	—	—	—	—	—	—	—	—	—	—
78	Toluene	—	—	—	—	—	—	—	—	—	—
79	Octanoic acid	2730.00	151	101	210	190	4550.00	91	60	126	114
80	Hexanoic acid	0.60	258,687	198,095	474,974	419,423	0.56	279,058	213,694	512,378	452,452
81	Acetic acid	23,078.00	<1	<1	<1	<1	74,479.00	<1	<1	<1	<1
82	3-Methylbutanoic acid	1110.00	17	27	31	23	462.50	40	64	75	54
83	Decanoic acid	8930.00	8	9	14	22	4554.30	16	18	27	42
84	Dodecanoic acid	10,000.00	9	6	15	12	529.80	172	121	277	221
85	Elaidic acid	—	—	—	—	—	—	—	—	—	—
86	DL-3-Methylvaleric acid	42.78	—	183	—	—	9.30	—	843	—	—
87	Heptanoic acid	587.52	11	—	11	9	2111.40	3	—	3	3
88	Tridecanoic acid	9582.00	<1	<1	<1	<1	—	—	—	—	—
89	2-Methylvaleric acid	—	—	—	—	—	11,868,480.00	<1	<1	<1	<1
90	Pentanoic acid	15,963.00	13	7	20	20	469.50	426	247	682	684
91	Nonanoic acid	4167.60	—	—	—	<1	1359.00	—	—	—	<1
92	Palmitic acid	8520.00	5	28	7	11	—	—	—	—	—
93	trans-3-Hexenoic acid	—	—	—	—	—	—	—	—	—	—
94	4-Methyl-2-pentenoic acid	—	—	—	—	—	—	—	—	—	—
95	Hendecanoic acid	8900.00	<1	—	<1	<1	890.00	5	—	3	2
96	Crotonic acid	—	—	—	—	—	—	—	—	—	—
97	2-Methyl-2-pentenoic acid	—	—	—	—	—	—	—	—	—	—
98	Furfuryl alcohol	5108.10	3	2	5	4	1135.00	14	9	24	18
99	2-Furancarboxylic acid	—	—	—	—	—	23,832.00	<1	<1	—	<1
100	2-Butylfuran	4.43	—	—	708	—	—	—	—	—	—
101	Furfural	3480.00	<1	1	2	2	9280.00	<1	<1	<1	<1
102	2-Acetyl furan	16,498.00	<1	<1	<1	<1	87,840.00	<1	<1	<1	<1
103	Methyl2-furoate	—	—	—	—	—	—	—	—	—	—
104	5-Hydroxymethyl-2-furaldehyde	1,243,000.00	<1	<1	<1	<1	1,243,000.00	<1	<1	<1	<1
105	2,5-Dimethyl-4-hydroxy-2H-furan-3-one	167.84	37	245	37	48	10.49	592	3926	600	772
106	2(5H)-Furanone	—	—	—	—	—	—	—	—	—	—
107	Furfuryl sulfide	—	—	—	—	—	—	—	—	—	—
108	2,2′-Difurfuryl ether	—	—	—	—	—	—	—	—	—	—
109	(±)-3-Hydroxy-4-butanolide	—	—	—	—	—	—	—	—	—	—
110	γ-Decanolactone	2.46	1192	1474	2091	3559	379.20	8	10	14	23
111	γ-Butyrolactone	1120.00	5	6	20	22	11,200.00	<1	<1	2	2
112	4-Nonanolide	26.35	265	164	272	389	976.00	7	4	7	11
113	γ-Hexanolactone	12,525.00	<1	<1	<1	<1	13,026.00	<1	<1	<1	<1
114	γ-Octalactone	17.56	480	271	509	659	93.20	90	51	96	124
115	δ-Valerolactone	—	—	—	—	—	1,105,000.00	<1	<1	<1	<1
116	γ-Valerolactone	10,000.00	<1	<1	<1	<1	105,000.00	<1	<1	<1	<1
117	Costunolide	—	—	—	—	—	—	—	—	—	—
118	5-Hydroxy-4-pentanolide	—	—	—	—	—	—	—	—	—	—
119	2-Hydroxypyridine	—	—	—	—	—	—	—	—	—	—
120	(Z)-9-Octadecenamide	—	—	—	—	—	—	—	—	—	—
121	2-Acetyl pyrrole	—	—	—	—	—	111,430.00	<1	<1	<1	<1
122	N-Butyl-acetamide	—	—	—	—	—	—	—	—	—	—
123	2,6-Di-tert-butylpyridine	—	—	—	—	—	—	—	—	—	—
124	5-Methyl-1H-pyrrole-2-carbaldehyde	—	—	—	—	—	—	—	—	—	—
125	6-(Ethoxycarbonyl)nicotinic acid	—	—	—	—	—	—	—	—	—	—
126	Benzylamine	2,943,000.00	<1	—	—	<1	209,934.00	<1	<1	<1	<1
127	Diethylamine	14,989.00	<1	—	—	<1	7070.00	<1	<1	<1	<1
128	3-Methylthiopropanol	71.07	349	345	563	383	2060.00	12	12	19	13
129	Thiophene	88,284.00	1	1	1	1	8.41	12,028	15,518	13,595	15,080
130	Methyl mercaptan	0.17	6794	1432	6750	9059	1.73	679	143	675	906
131	Dimethyl Sulfoxide	—	—	—	—	—	—	—	—	—	—
132	Dimethyl sulfone	—	—	—	—	—	—	—	—	—	—
133	Thioacetic acid	100.00	183	—	—	105	—	—	—	—	—
134	Tetrahydrothiophene	—	—	—	—	—	—	—	—	—	—
135	2-Acetyl-2-thiazoline	—	—	—	—	—	1.17	—	70,957	—	—
136	4-Methyl-5-acetyl thiazole	—	—	—	—	—	—	—	—	—	—
137	(E)-2-Nonenal	0.16	149,938	41,011	—	—	0.21	113,953	31,168	—	—
138	Isovaleraldehyde	0.95	8845	—	—	—	36.39	231	—	—	—
139	Acetaldehyde	49.46	53	33	17	15	7850.00	<1	<1	<1	<1
140	Hexadecanal	—	—	—	—	—	—	—	—	—	—
141	4-Hydroxy-2-butanone	—	—	—	—	—	—	—	—	—	—
142	(Z)-β-Ionone	0.01	—	14,243,050	—	12,784,795	—	—	—	—	—
143	3-Hydroxy-2-butanone	14.18	5325	4822	2838	5525	17,221.00	4	4	2	5
144	Cholestane	—	—	—	—	—	—	—	—	—	—
145	1-Heptene	1045.50	478	441	578	489	—	—	—	—	—
146	1-Undecene	—	—	—	—	—	—	—	—	—	—
147	2-Methyl-2-butene	—	—	—	—	—	—	—	—	—	—
148	Myrcene	3.88	1083	—	962	—	13.13	320	—	284	—
149	Diisopropyl ether	—	—	—	—	—	—	—	—	—	—
150	3-Methyl-1-butene	—	—	—	—	—	—	—	—	—	—
